# Dysregulated *paired related homeobox 1* impacts on hepatocellular carcinoma phenotypes

**DOI:** 10.1186/s12885-021-08637-3

**Published:** 2021-09-08

**Authors:** Weronika Piorońska, Zeribe Chike Nwosu, Mei Han, Michael Büttner, Matthias Philip Ebert, Steven Dooley, Christoph Meyer

**Affiliations:** 1 Department of Medicine II, Section Molecular Hepatology, Medical Faculty Mannheim, Heidelberg University, Theodor-Kutzer-Ufer 1–3, 68167 Mannheim, Germany; 2 Current address: Department of Molecular and Integrative Physiology, Rogel Cancer Centre, University of Michigan, Ann Arbor, MI 48109 USA; 3 Current address: Department of Internal Medicine, the Second Hospital of Dalian Medical University, Shahekou District, Dalian City, Liaoning Province China; 4 The Metabolomics Core Technology Platform of the University of Heidelberg, Im Neuenheimer Feld 360, 69120 Heidelberg, Germany

**Keywords:** EMT, Epithelial mesenchymal transition, Liver cancer, Metabolism, PRRX1

## Abstract

**Background:**

Hepatocellular carcinoma (HCC) is a major cause of cancer-related death. Paired related homeobox 1 (PRRX1) is a transcription factor that regulates cell growth and differentiation, but its importance in HCC is unclear.

**Methods:**

We examined the expression pattern of *PRRX1* in nine microarray datasets of human HCC tumour samples (*n* > 1100) and analyzed its function in HCC cell lines. In addition, we performed gene set enrichment, Kaplan-Meier overall survival analysis, metabolomics and functional assays.

**Results:**

*PRRX1* is frequently upregulated in human HCC. Pathway enrichment analysis predicted a direct correlation between *PRRX1* and focal adhesion and epithelial-mesenchymal transition. High expression of *PRRX1* and low *ZEB1* or high *ZEB2* significantly predicted better overall survival in HCC patients. In contrast, metabolic processes correlated inversely and transcriptional analyses revealed that glycolysis, TCA cycle and amino acid metabolism were affected. These findings were confirmed by metabolomics analysis. At the phenotypic level, *PRRX1* knockdown accelerated proliferation and clonogenicity in HCC cell lines.

**Conclusions:**

Our results suggest that *PRRX1* controls metabolism, has a tumour suppressive role, and may function in cooperation with *ZEB1/2*. These findings have functional relevance in HCC, including in understanding transcriptional control of distinct cancer hallmarks.

**Supplementary Information:**

The online version contains supplementary material available at 10.1186/s12885-021-08637-3.

## Background

Liver cancer is a common and deadly cancer of which the most prevalent type is hepatocellular carcinoma (HCC) [1]. Recent advances in molecular profiling have highlighted the importance of gene deregulation in hepatocellular carcinogenesis [2]. Accordingly, several potential molecular drivers of HCC have been identified, including mutations in *TP53, CTNNB1,* and *TERT* promoter [3], and metabolic genes such as *ALB*, *CPS1,* and *APOB* [4]. However, molecular heterogeneity and the main mechanisms promoting HCC are poorly understood.

Transcription factors are known to control key processes in cancer progression such as growth, metabolism, immune evasion, and metastasis [5, 6]. The paired related homeobox 1 (PRRX1) is a transcriptional co-activator that exist in two isoforms. The functions of *PRRX1* include the regulation of cell growth and differentiation. Consistently, *Prrx1* deletion is lethal in neonatal mice [7]. *PRRX1* has been found to be overexpressed in breast, pancreas, head and neck squamous cell carcinoma, liver, and colon cancer [8–14]. In murine pancreatic cancer and human breast cancer cell lines, the knockdown of the isoforms *PRRX1a* or *PRRX1b* reduced migration and invasion, indicating functional similarities [9, 14, 15]. However, overexpression of *PRRX1a* led to the induction of genes involved in cell migration whereas *PRRX1b* was more involved in cell cycle regulation [9], supporting that these isoforms could have distinct molecular functions. The knockdown of *PRRX1b* inhibited proliferation as well as migratory and invasive capabilities of triple negative breast cancer cell lines [14]. In other studies, knockdown of *PRRX1* reduced tumour volume of MDA-MB-231 mice xenografts [14], and its overexpression in colon cancer cell lines increased colony formation and anchorage independent growth [10]. In the HCC cell line HUH7, ectopic expression of *PRRX1* induced resistance to 5-fluoruracil [12]. These data indicate a tumour-promoting role of *PRRX1* in some cancer settings. On the contrary, *PRRX1* has also been shown to exert tumour suppressor functions. For example, low expression of *PRRX1* enabled metastatic colonization of lung by breast cancer cells [8]. HCC cells were also shown to migrate more upon *PRRX1* knockdown [13]. In clinical contexts, *PRRX1* has been associated to contradictory prognostic outcomes showing that high *PRRX1* expression predicted improved overall survival in colorectal cancer and HCC [10, 12, 13], but was associated with metastasis and poor survival outcome in breast cancer [8].

Although the precise roles of *PRRX1* and its isoforms, *PRRX1a* and *PRRX1b,* are unclear in human cancer, *PRRX1* was often associated with cancer stemness and epithelial-mesenchymal transition (EMT) [8, 9, 15]. For example, the overexpression of *PRRX1* in human colon cancer cell lines induced EPH receptor B2 – an intestinal stem cell marker [10], whereas the knockdown of *PRRX1* in breast cancer increased stemness features [8]. Regarding EMT, overexpression of *PRRX1* caused the upregulation of the EMT gene *TWIST1* [8]. In addition, its knockdown reversed invasiveness [15]. Few studies have investigated *PRRX1* expression in HCC [12, 13, 16], and its functions are largely unknown. Based on the contradictory reports on *PRRX1*, including on expression level in HCC, we set out to comprehensively analyze its expression and to predict its functions in human HCC. Making use of several HCC tissue gene expression datasets and applying functional assays in vitro*,* we find that *PRRX1* is frequently upregulated in human HCC. We identified two EMT transcriptional factors, zinc finger E-box-binding homeobox (*ZEB1* and *ZEB2*) as novel *PRRX1*-related genes, and report that *PRRX1* knockdown affects the phenotype of HCC cell lines, including metabolism.

## Materials and methods

### Collection of liver cancer microarray datasets and analyses

Eight liver cancer microarray datasets were obtained from the National Center for Biotechnology Information Gene Expression Omnibus (NCBI GEO), whereas the TCGA liver cancer data was accessed via cBioPortal platform (http://www.cbioportal.org/) [17, 18] and http://cancergenome.nih.gov. The expression values for *PRRX1* probes were compiled for each dataset, and its differential expression in HCC compared to normal or adjacent non-tumour tissues was determined using Student *T-test* in GraphPad Prism version 6. Genes positively or negatively co-expressed with *PRRX1* in liver cancer data were downloaded from cBioPortal platform and used for correlation analyses in TCGA and GSE14520 datasets. For *PRRX1*-high versus low expressing tumour comparison, TCGA and GSE14520 samples were ranked based on *PRRX1* expression level and divided into two groups (based on median). The high (*n* = 186) vs low (*n* = 185) samples from TCGA were subsequently compared in R, using limma package. For Kaplan-Meier overall survival analysis of *PRRX1* in combination of other genes, we first considered whether the genes have significant direct or inverse correlation with *PRRX1* prior to subsetting samples for comparison. For example, for genes that met the criteria of direct correlation with *PRRX1*, the tumours with high expression of the gene and *PRRX1* were compared with tumours with low expression of both. In contrast, for genes inversely correlated with *PRRX1*, tumours with high *PRRX1* (*n* = 111) and low expression of the genes were selected and compared with tumours with low *PRRX1* (*n* = 110) and high level of the gene. Analysis of cancer hallmark pathways was done with the ‘GSEAPreranked’ option in GSEA (http://software.broadinstitute.org/gsea/index.jsp).

### Cell culture

The Hep3B, HLF and HUH7 cell lines were provided by Prof Kern (Pathology, Heidelberg). The SNU398 cells were obtained from Dr. Francois Helle (University of Picardie Jules Verne, France). The cell lines HLF, HUH7, Hep3B and SNU398 were cultured in Dulbecco Modified Eagle’s medium (DMEM, high glucose, Lonza, BE12–709) supplemented with 2 mM glutamine, 10% fetal bovine serum (FBS), penicillin (100 U/ml), and streptomycin (100 μg/ml). The cells were cultured at 37 °C in a humidified atmosphere containing 5% CO_2_. Hank’s Balanced Salt Solution (HBSS) (Sigma-Aldrich, H6648) was used for cell washing steps. The cells were used for experiments between passage 2-10. All used cell lines were authenticated by short tandem repeat profiling (STR). PCR Mycoplasma Test (PromoCell, Huissen, Netherlands) was performed to confirm that cells are mycoplasma-free. siRNA transfection and RNA isolation were performed as described in the supplement.

### Quantitative PCR

Quantitative polymerase chain reaction (qPCR) was performed using EvaGreen qPCR Mix Plus (Solis BioDyne, Tartu, Estonia) on the AB StepOnePlus. The experiments were performed in triplicates using peptidylprolyl isomerase A (*PPIA*) as control. The primers (Table 1) were ordered from Eurofins Genomics (Ebersberg, Germany). *PRRX1* expression was evaluated in liver tissue (*n* ≥ 4) from MDR2 knockout mice on a 129 background (3, 6, 9, and 15 months) and age matched controls.

**Table 1:** Primer list

	Forward (5′– 3′)	Reverse (5′– 3′)
h*FH*	CGTTTTGGCCTCCGAACG	CATGCGTTCTGTCACACCTC
h*GOT1*	CAACTGGGATTGACCCAACT	GGAACAGAAACCGGTGCTT
h*GOT2*	TAACGTTCTGCCTAGCGTCC	ACTTCGCTGTTCTCACCCAG
h*GLS1*	GCAACAGCGAGGGCAAAGAG	CTGGGATCAGACGTTCGCAAT
h*GPT1*	GGTCTTGGCCCTCTGTGTTA	TCCGCCCTTTTCTTGGCATC
h*GPT2*	GACCCCGACAACATCTACCTG	TCATCACACCTGTCCGTGACT
h*HK1*	CCAACATTCGTAAGGTCCATTCC	CCTCGGACTCCATGTGAACATT
h*HK2*	CCAGATGGGACAGAACACGG	TGGAGCCCATTGTCCGTTAC
h*IDH3A*	ATCGGAGGTCTCGGTGTG	AGGAGGGCTGTGGGATTC
h*IDH3B*	TCTCAGCGGATTGCAAAGTTTG	CTTGTGGACAGCTGTGACCTT
h*LDHA*	GCAGCCTTTTCCTTAGAACAC	AGATGTTCACGTTACGCTGG
h*LDHB*	CTTGCTCTTGTGGATGTTTTGG	TCTTAGAATTGGCGGTCACAG
h*MDH1*	CATTCTTGTGGGCTCCATGC	AGGCAGTTGGTATTGGCTGG
h*OGDH*	GAGGCTGTCATGTACGTGTGCA	TACATGAGCGGCTGCGTGAACA
h*PCK1*	GCAAGACGGTTATCGTCACCC	GGCATTGAACGCTTTCTCAAAT
h*PDHX*	TTGGGAGGTTCCGAC	CAACCACTCGACTGTCACTTG
h*PPIA*	AGGGTTCCTGCTTTCACAGA	CAGGACCCGTATGCTTTAGG
m*Ppia*	GAGCTGTTTGCAGACAAAGTT	CCCTGGCACATGAATCCTGG
h*PRRX1*	GAAGAGAAAGCAGCGAAGGA	ACTTGGCTCTTCGGTTCTGA
h*PRRX1a*	CGAGAGTGCAGGTGTGGTTT	AATCCGTTATGAAGCCCCTCG
h*PRRX1b*	GTCTCCGTACAGCGCCAT	GGCCTTCAGTCTCAGGTTGG
m*Prrx1*	AAGCAGCGGAGAAACAGGAC	ACAAAAGCATCCGGGTAATGTG
h*SDHA*	TGATGGGAACAAGAGGGCATC	ACCTGGTAGGAAACAGCTTGG
h*SDHB*	CACCCGAAGGATTGACACCA	GTTGCTCAAATCGGGAACAAGA
h*SDHC*	TCCTCTGTCTCCCCACATTACT	CCAGACACAGGGACTTCACAA
h*SDHD*	GCAGCACATACACTTGTCACC	CTGACAACCCTCTCGCTAGTC
h*SLC1A5*	TTTGCGGGTGAAGAGGAAGT	AGCATTCCGAAACAGGTAACTTT
h*SUCLG1*	ATTATGCCGGGTTACATCCA	AAAAGGATCCCCACCAATTC
h*SUCLG2*	TTTGCTATGGACGACAAATCAGA	CTGGCTTCCCACCATTAAGG
h*ZEB1*	CAGCTTGATACCTGTGAATGGG	TATCTGTGGTCGTGTGGGACT
h*ZEB2*	GGAGACGAGTCCAGCTAGTGT	CCACTCCACCCTCCCTTATTTC

*h* human, *m* mouse

### MTT proliferation assay

Proliferation assays were performed with 3-(4,5-dimethylthiazol-2-yl)-2,5-diphenyltetrazolium bromide (MTT) assay. Briefly, 7.5–10 × 10^3^ cells per well were seeded in quadruplicates in 48-well plates. The cells were incubated o/n and thereafter transfected with the respective siRNA oligos. After the indicated time, 25 μl of 5 mg/ml MTT reagent (Sigma Aldrich, USA) was added to the wells and incubated for 3–4 h at 37 °C. The media was subsequently aspirated and 250 μl of MTT solubilizing reagent was added to dissolve the formed formazan crystals. The plate was then incubated o/n at 37 °C. Absorbance was read at 560 nm with background correction at 670 nm using Infinite 200 Spectrophotometer (Tecan, Austria) and the data were normalized to the control wells.

### Clonogenic assay

The HCC cells were initially seeded into 12 well plates and allowed to attach overnight. Culture medium was then replaced with medium containing siRNA/transfection reagent. After 24 h, the cells were trypsinized and seeded in triplicates in 6-well plates (2500 per well). Medium was changed at day 4 and the experiment terminated at day 8. Cells were fixed for 5 min with methanol (Carl Roth, Karlsruhe, Germany) and stained for 15 min in 0.5% crystal violet (Alfa Aesar, Karlsruhe, Germany). The wells were then washed with running tap water, allowed to dry and photographed.

### In vitro scratch assay

5 × 10^5^ cells were seeded in triplicate into 12 well plates (Greiner Bio-One, 665,180), and incubated o/n, followed by siRNA transfection. Next day the wells were scratched with 200 μl pipette tips and reference points made using a needle. Images were taken from the wells at time points 0 and 24 h later using an inverted microscope (Leica, Wetzlar, Germany). ImageJ was used to measure the distance/gap between the two edges of the scratch. Migration was calculated as difference between gap distance at time 0 and 24 h divided by the distance at time 0.

### Measurement of glucose consumption and lactate output

Cells (1.5 × 10^5^) were seeded in triplicate into 12 well plates and allowed to attach o/n. Then, siRNA transfection was performed. 48 h after transfection, cell culture medium was collected and analyzed for glucose and lactate using the Roche Cobas C311 Chemistry Analyzer. Cells were lysed with RIPA buffer and data was normalized to protein concentration.

### Metabolite analyses

One million of HUH7 and HLF cells were seeded in triplicate in 14.5 cm petri dishes and allowed to attach o/n. The next day, siRNA transfection was performed. After 24 h, medium was changed to growth medium for another 48 h. Thereafter, medium was removed, cells were washed with pre-warmed pure water and immediately snap frozen by adding liquid nitrogen to the petri dishes. Extraction of the samples was performed as follows: frozen HUH7 and HLF cells were extracted directly on petri dishes by adding pre-cooled 1 ml 50% methanol and 10 μl Ribitol (0.2 mg/ml; internal standard for the polar phase). All liquid containing cell debris was transferred to 2 ml reaction tubes on ice. To each tube, 0.5 ml 100% chloroform containing 0.1 mg/ml heptadecanoic acid (internal standard for organic phase) was added and samples were vortexed for 10 s. To separate polar and organic phases, as described previously [19, 20], samples were centrifuged for 10 min at 11,000x g. For derivatization, 0.9 ml of the upper polar phase were transferred to a fresh tube and speed-vac dried without heating. Pellets of the aqueous phase after extraction were dissolved in 20 μl methoximation reagent containing 20 mg/ml methoxyamine hydrochloride in pyridine and incubated for 2 h at 37 °C under vigorous shaking. For silylation, 35 μl N-methyl-N-(trimethylsilyl) trifluoroacetamide were added to each sample. After incubation for 45 min at 50 °C, samples were analyzed by Gas Chromatography/Mass Spectrometry (GC/MS). A GC/MS-QP2010 Plus (Shimadzu, Germany) fitted with a Zebron ZB 5MS column (Phenomenex; 30 m × 0.25 mm × 0.25 μm) was used for GC/MS analysis. The GC was operating with an injection temperature of 250 °C and 1 μl sample was injected with split mode (diluted 1:5). The GC temperature program for polar compounds started with 1 min hold at 40 °C followed by a 6 °C/min ramp to 210 °C, a 20 °C/min ramp to 330 °C and a bake-out for 5 min at 330 °C. Helium was used as carrier gas with consistent linear velocity. The MS was operated with ion source and interface temperatures at 250 °C, a solvent cut time of 5 min and a scan range (m/z) of 40–700 with an event time of 0.1 s. Raw data were processed using the „GCMS solution software” (Shimadzu) and normalized to the internal standard Ribitol as well as to the cell number.

### Statistics

Results are presented as mean ± SD unless indicated otherwise. Comparison of sample groups was performed using GraphPad Prism version 6 Software or R statistical software. Where applicable, t-test was applied for unpaired sample comparison, while one-way ANOVA was used for multiple comparisons. Statistical significance was defined with *P* < 0.05. Quantification of migration was done using ImageJ 1.5 (http:imagej.nih.gov/ij). Statistical significance is indicated as follows: * *P* < 0.05, ** *P* < 0.01, *** *P* < 0.001, and **** *P* < 0.0001.

## Results

### PRRX1 is frequently upregulated in HCC

**Fig. 1:**
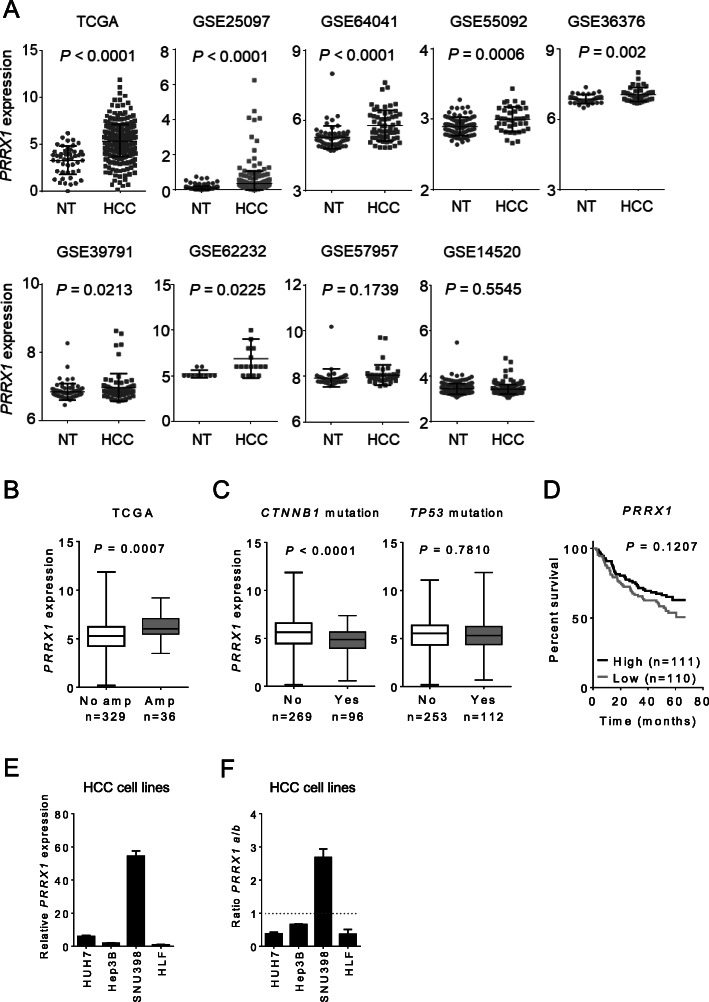
*PRRX1* is upregulated in human HCC and shows a variable pattern in vitro*.* **A** *PRRX1* expression in human HCC cohorts. NT - non-tumour, HCC - hepatocellular carcinoma. Data was analysed with Student’s t-test. Information about sample size per cohort is contained in Table S1. **B** *PRRX1* expression in tumours showing its gene amplification. Data was analysed with Student’s t-test. Amp: amplification **C** *PRRX1* expression in tumours with *TP53* and *CTNNB1* mutation in TCGA liver cancer cohort. Data was analysed with Student’s t-test. **D** Kaplan-Meier overall survival analysis (logrank test) based on *PRRX1* expression in GSE14520 dataset. High *PRRX1 n* = 110; low *PRRX1 n* = 110. **E** *PRRX1* expression in human HCC cell lines after 48 h culture. Normalized to the basal expression in HLF (lowest *PRRX1* level). Bars indicate mean ± SD, *n* = 3 per group. **F** Ratio of PRRX1a to PRRX1b isoform expression in HCC cell lines after 48 h culture. Bars indicate mean ± SD, *n* = 3 per group

We analyzed *PRRX1* expression in nine human HCC gene expression datasets (Table S1). Out of these datasets, *PRRX1* level were upregulated in seven (*P* < 0.05), and not significantly changed in two datasets (Fig. 1a). In addition, we compared the expression of *PRRX1* across liver cancer cohorts in Oncomine – an online repository of manually curated cancer datasets (http://oncomine.org). The Oncomine platform contains the cancer genome atlas (TCGA) liver cancer data and GSE14520 dataset used in our analysis, thus was also advantageous for cross-validating our observation. Indeed, in Oncomine platform *PRRX1* was upregulated in three datasets, but not significantly changed in five other datasets (Fig. S1A). Hence, we concluded that when significantly altered, *PRRX1* is upregulated in HCC.

We then compared the alteration frequency of *PRRX1* in TCGA cancer datasets via cBioPortal (http://cbioportal.org). *PRRX1* alterations in liver cancer (i.e. HCC and cholangiocarcinoma) along with its alterations in lung, bladder and breast cancer cohorts, ranked very high (within top 10% of all TCGA cohorts) (Fig. S1B). Of note, *PRRX1* was amplified in ~ 10% (*n* = 36) of TCGA HCC tumour samples, but this amplification did not strikingly overlap with known mutated genes in HCC, i.e. *TP53* or *CTNNB1* (Fig. S1C). As expected, HCC tumours with high amplification showed higher mRNA expression (*P* = 0.0007) compared to the tumours with no amplification (Fig. 1b). Further, we compared *PRRX1* expression based on two frequently mutated genes in HCC (i.e. *TP53* and *CTNNB1*). Accordingly, tumours with *CTNNB1* mutation showed low *PRRX1* level (*P* < 0.0001), whereas no expression change was observed with *TP53* mutations (Fig. 1c), suggesting that the different oncogenic background influences *PRRX1* level.

A prior study using the GSE14520 dataset showed an improved OS with high *PRRX1* expression [12]. To gain insight on association of *PRRX1* with clinicopathological variables, we analyzed clinical data from one of the two platforms used in GSE14520 (GPL3921, *n* = 225 HCC samples). Upon Kaplan-Meier overall survival (OS) analysis, we found a tendency towards improved outcome for patients with high *PRRX1*-expressing tumours, but this was not statistically significant (Fig. 1d). Also, *PRRX1* was not associated with other clinical variables analyzed, e.g. tumour size, stage, alanine transaminase (ALT) and alpha-fetoprotein (AFP) level (Fig. S2A).

We further analyzed *PRRX1* expression patterns in experimental HCC models. In multi-drug resistance (*Mdr2*) knockout mice – a liver fibrosis model that progresses to HCC in about 12 months [21, 22] – we observed higher *Prrx1* expression in *Mdr2* knockout mice than in age - matched controls and a lower expression of *Prrx1* in older mice with HCC compared to the younger *Mdr* mice (Figure S2B).

In HCC cell lines, *PRRX1* expression was variable. Specifically, it was highest in SNU398, a cell line with epithelial-like appearance albeit reported to be poorly differentiated [23]. Other cell lines with epithelial features, HUH7 and Hep3B, showed higher *PRRX1* level when compared to HLF cells that display more mesenchymal properties (Fig. 1e). In these four cell lines, we additionally analyzed expression of *PRRX1* isoforms, a and b. With the exception of SNU398, the HCC cell lines (Fig. 1f) expressed less of the truncated isoform (i.e. *PRRX1a*) than the longer isoform *PRRX1b*. These analyses support that *PRRX1* is frequently upregulated in liver tumours, while data from experimental models suggest that *PRRX1* may be more expressed in well differentiated cells.

### PRRX1 correlates directly with cancer pathways and inversely with metabolic pathways

**Fig. 2:**
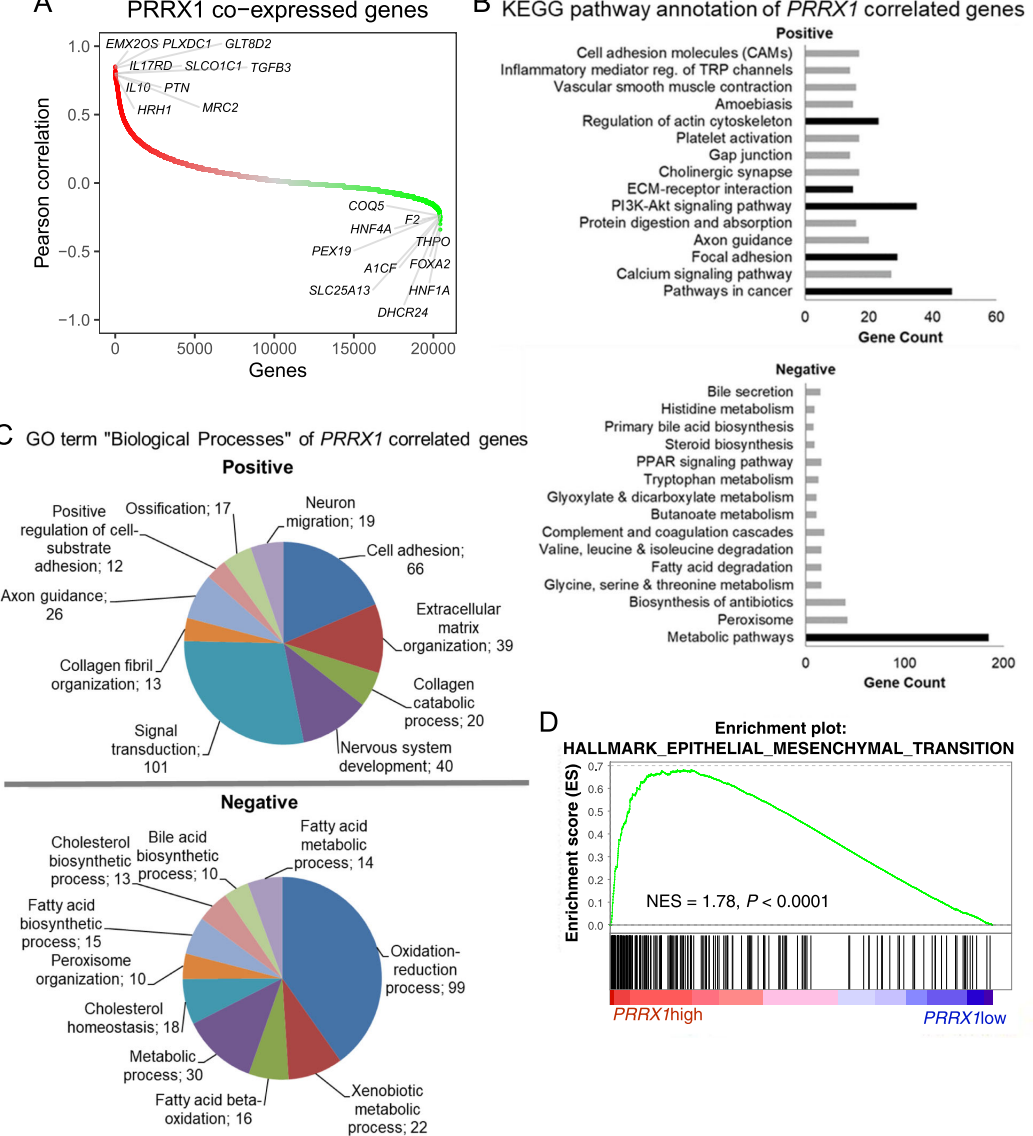
Functional enrichment analyses of *PRRX1* positively and negatively co-expressed genes. **A** *PRRX1* positively and negatively correlated genes in TCGA liver cancer data. PRRX1-correlated genes were obtained from cBioPortal platform (http://www.cbioportal.org/). **B** KEGG pathway annotation of genes positively and negatively correlated with *PRRX1*. **C** Gene ontology of ‘biological process’ of *PRRX1*-correlated genes. **D** GSEA plot showing enrichment of epithelial–mesenchymal transition in the genes positively correlated with *PRRX1*. NES – normalized enrichment score

To predict the functions or pathways associated with the expression of *PRRX1*, we first identified *PRRX1* co-expressed genes in TCGA HCC data from the cBioPortal platform (Table S2). When ranked based on Pearson score, the topmost *PRRX1*-co-expressed genes included several known candidates in HCC, e.g. *TGFB3, HNF1A, IL10* [3] as well as novel targets such as *PLXDC1, EMX2OS,* and *GLT8D2* (Fig. 2a). Functional annotation analyses using the top positively correlated genes (*n* = 1022, ~ 5% of the gene list), indicated an overrepresentation of cancer processes, i.e., extracellular matrix (ECM) and signal transduction activities (Fig. 2b). Specific processes that emerged include ECM-receptor interaction (*n* = 15 genes, e.g., *COL6A1/A2/A3, ITGA8/10, LAMA2/4*), focal adhesion (*n* = 29 genes, e.g.*. CAV1, PDGFRA, PDGFRB, TNC, COL1A1*), and PI3K-AKT signaling (*n* = 35 genes, e.g. *PDGFRA/RB, FGF1/9, FGFR1, JAK2, LPAR1/4/5/6, GNG2, PIK3R3/R5*) (Tables S3 and S4). Consistently, gene ontology (GO) analyses for biological processes and cellular components supported a positive correlation with cancer processes, notably ECM organization, cell adhesion and signal transduction, as mentioned earlier (Fig. 2c, Fig. S3, and Tables S5 and S6). Unsurprisingly, gene set enrichment analysis (GSEA) of *PRRX1* co-expressed genes also identified EMT (Fig. 2d). Further, the GO class ‘cellular component’ contained 293 genes clustered to plasma membrane, and those included *MMP2, CAV1, FGFR1, PLPPR4*, *TGFB3*, *MSR1*, and transporters such as *SLC1A5, SLC6A6, SCN3A* and *SLC7A3.* Similarly, > 300 genes were assigned as integral membrane components (Fig. S3), altogether implicating *PRRX1* in membrane dynamics, cell plasticity and molecule transport. Other cancer types that showed high *PRRX1* alteration frequency in the TCGA cohorts (e.g., cholangiocarcinoma and lung cancer), also showed similar pathway annotation patterns as observed in HCC (i.e. focal adhesion and ECM organization, Fig. S4).

The top *PRRX1*-negatively correlated genes (*n* = 1022) were mostly involved in metabolism, a process that has not yet been associated with *PRRX1* in HCC (Fig. 2b and c). We found that prominent inversely correlated metabolic processes include glycine, serine and threonine metabolism (*n* = 16 genes, e.g., *SHMT1, CHDH, GATM*), fatty acid degradation (*n* = 16 genes, e.g., *ECI1, ECI2, ACOX1*), valine, leucine and isoleucine degradation (*n* = 16 genes, e.g. *ACADSB, EHHADH, ECHS1*), and peroxisome activity (*n* = 42 genes, e.g. *ACOX1/2, HACL1, PEX1, 5, 6, 7, 16, and 19*) (Fig. 2b). Metabolism also dominated the GO term ‘biological processes’ of the negatively correlated genes (Fig. 2c, Table S7). Further, the GO term ‘cellular component’ implicated peroxisomes and mitochondria (Fig. S3, Table S8) – two organelles involved in antioxidant defenses and cellular respiration, respectively. Given the link to metabolism, we wondered if the *PRRX1* inversely correlated genes included consistently altered metabolic genes in human HCC [24]. Indeed, overlap of all *PRRX1*-correlated genes with metabolic genes from human HCC revealed 135 common elements (Table S9, Fig. S5A). Of these, 124 metabolic genes were downregulated in HCC and several of them belonged to amino acid and fatty acid metabolism, and also small molecule transport (Fig. S5B). Thus, *PRRX1* expression correlated with the downregulation of metabolic pathways in HCC. In cholangiocarcinoma and lung cancer, we also observed a negative correlation with metabolic pathways, suggesting that similar functions predicted for *PRRX1* in HCC may apply to other cancer types (Fig. S4). Taken together, these data suggest that *PRRX1* promotes cancer pathways and contributes to a suppressed metabolic gene program, as previously seen in HCC [4, 24, 25].

### Integrative analyses identify EMT genes ZEB1 and ZEB2 as novel transcription factors related to PRRX1

**Fig. 3:**
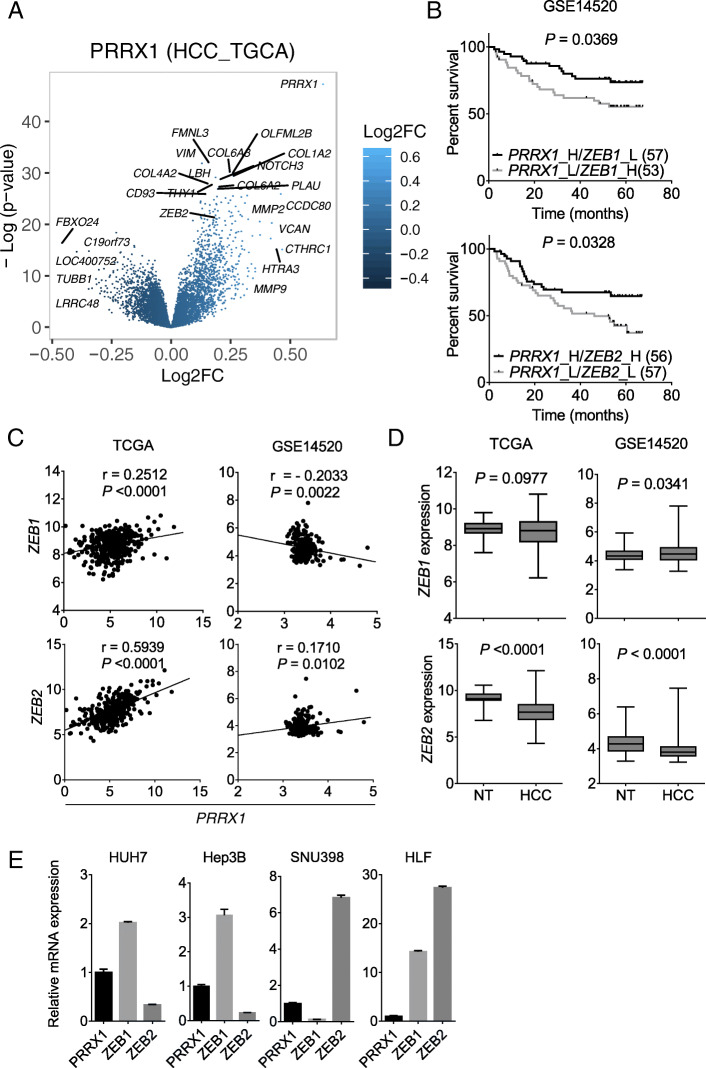
*ZEB1* and *ZEB2* are novel *PRRX1* related genes. **A** Volcano plot showing genes differentially expressed in *PRRX1*-high tumours from TCGA dataset. **B** Kaplan Meier OS analysis (logrank test) of high *PRRX1* combined with low *ZEB1* or high *ZEB2* in GSE14520 dataset, based on its negative correlation with *ZEB1* and positive correlation with *ZEB2* in this dataset. H = high, L = low. **C** Pearson correlation of *PRRX1* and *ZEB1* or *ZEB2* in two HCC collectives. **D** *ZEB1* and *ZEB2* expression in two representative HCC cohorts. Data was analysed with Student’s t-test. NT – non-tumour. TCGA: NT *n* = 50, HCC *n* = 373; GSE14520: NT *n* = 220, HCC *n* = 225. (E) Differential expression of *ZEB1* and *ZEB2* compared to *PRRX1* in HCC cell lines. Cells were cultured for 48 h prior to analysis, bars indicate mean ± SD, *n* = 3 per group

Based on our in silico analyses of several HCC patient gene expression datasets, which linked *PRRX1* to metabolism and cancer pathways, we first focused on its influence towards cellular plasticity and EMT. Thus, we stratified TCGA HCC dataset into high versus low *PRRX1*-expressing tumour samples, and analyzed the differential gene expression. We observed that the *PRRX1*-high tumours expressed several cancer-associated genes (e.g. *MMP2/9, ZEB2, VIM*) (Fig. 3a, Table S10). This expression pattern was consistent with that of the co-expressed gene list from cBioPortal (which generally reflected EMT, ECM, cancer and metabolic pathway alterations as earlier noted in Fig. 2). We compiled ~ 150 genes involved in these processes (Table S11), including the topmost PRRX1 co-expressed genes (Fig. 2a), and sought to identify those correlated with *PRRX1* in TCGA HCC data and another dataset (GSE14520). Of those genes, 19 significantly correlated with *PRRX1* in both datasets (Table 2). The EMT transcription factors *ZEB1* and *ZEB2* emerged alongside *HNF1A, IL10,* and 15 metabolic genes, e.g., gamma-glutamyltransferase 5 (GGT5), glucose transporter 2 (*SLC2A2*), NADPH oxidase 4 (*NOX4*), alcohol dehydrogenase 6 (*ADH6*), and hepatic lipase C (*LIPC*) (Fig. S6A). Kaplan-Meier OS analysis for each of the 19 genes in combination with *PRRX1* showed that liver tumours with high *PRRX1* and low *ZEB1 (P* = 0.0369*)*, high *ZEB2* (*P* = 0.0328), high *GGT5* (*P* = 0.0015) or high *SLC7A8* (*P* = 0.0016) showed improved OS outcome (Fig. 3b, Fig. S6B). *ZEB1* or *ZEB2* did not predict OS when analyzed alone (Fig. S7A) indicating a synergistic impact with *PRRX1*. Since *PRRX1* is associated with EMT [8, 9, 15], but no prior study had linked it with *ZEBs* in HCC, we performed further analysis on *ZEBs*.

**Table 2:** Top genes significantly correlated with *PRRX1* in human HCC

^a^Gene symbol	Gene name
*A1CF*	APOBEC1 complementation factor
*ACAA1*	acetyl-CoA acyltransferase 1
*ACOX2*	acyl-CoA oxidase 2
*ADH6*	alcohol dehydrogenase 6 (class V)
*CYB5A*	cytochrome b5 type A
*CYP2J2*	cytochrome P450 family 2 subfamily J member 2
*GGT5*	gamma-glutamyltransferase 5
*HNF1A*	HNF1 homeobox A
*IL10*	interleukin 10
*LIPC*	lipase C, hepatic type
*MGST2*	microsomal glutathione S-transferase 2
*MTTP*	microsomal triglyceride transfer protein
*NOX4*	NADPH oxidase 4
*PAH*	phenylalanine hydroxylase
*SLC2A2*	solute carrier family 2 member 2
*SLC7A8*	solute carrier family 7 member 8
*SORD*	sorbitol dehydrogenase
*ZEB1*	zinc finger E-box binding homeobox 1
*ZEB2*	zinc finger E-box binding homeobox 2

^a^These genes significantly correlated with *PRRX1* (*P* < 0.05) in two cohorts GSE14520 (*n* = 225 samples) and TCGA HCC (*n* = 371 samples). Pearson correlation analysis was used

Analyses of multiple HCC cohorts showed that both *ZEB1* and *ZEB2* expression positively correlated with *PRRX1* in TCGA, GSE25097 and GSE55092 datasets. In addition, *ZEB2* correlated positively with *PRRX1* in the dataset GSE64041. On the other hand, we found one dataset each (GSE14520 and GSE36376) where *ZEB1* and *ZEB2*, respectively, were negatively correlated with *PRRX1* (Fig. 3c, Fig. S7B). Thus, *PRRX1* is more often positively correlated with *ZEB1* and *ZEB2* in patients’ HCC tumour samples. However, besides OS, *PRRX1* combined with *ZEB1* or *ZEB2* expression was not significantly associated with several clinicopathological variables (Tables S12, S13). Next, we sought to determine whether *ZEB1* and *ZEB2* show a consistent expression pattern across the HCC cohorts. Out of nine datasets, we found that *ZEB1* was high in six while *ZEB2* was high in two datasets. *ZEB2* was significantly low in five datasets (Fig. 3d, Fig. S7C), thus indicating that *ZEB1* is generally high in HCC while *ZEB2* is often downregulated. We also analyzed *ZEB1*/*2* expression in tumours with *CTNNB1* and *TP53* mutations, which are frequent in HCC. We found that *ZEB1* is downregulated in *TP53-*mutated tumours and not changed in *CTNNB1* mutations, whereas *ZEB2* was upregulated in tumours with *CTNNB1* mutation and not changed in *TP53*-mutated tumours (Fig. S7D), further supporting that the *ZEB*s often have a contrasting expression pattern.

In cell lines, we measured the basal mRNA level of *ZEB1/2* relative to *PRRX1* and observed a heterogeneous expression pattern. Specifically, poorly differentiated cell line HLF, which expresses *PRRX1* at low level (Fig. 1e), showed comparatively higher levels of both *ZEB1/2* (Fig. 3e). SNU398 cells, which express high *PRRX1*, showed low *ZEB1* and high *ZEB2*, whereas HUH7 and Hep3B cells (well-differentiated cells) expressed higher *ZEB1* and lower *ZEB2* compared to *PRRX1* expression (Fig. 3e). Taken together, we identify *ZEB1* and *ZEB2* as EMT genes correlated with *PRRX1* and assumingly acting with it to influence tumour characteristics.

### Modulation of PRRX1 alters HCC cell phenotype

**Fig. 4:**
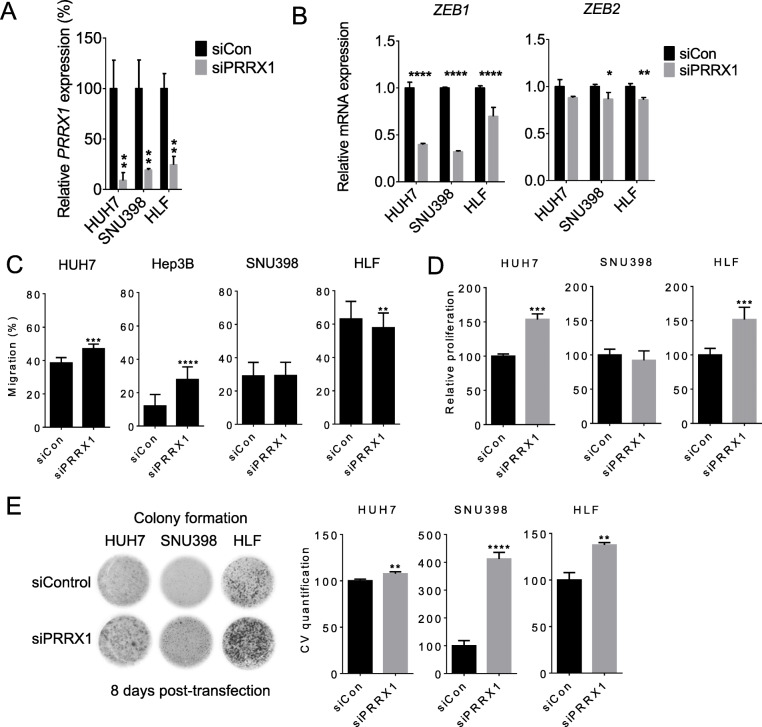
*PRRX1* acts as tumour suppressor in HCC cell lines and affects *ZEB1/2* expression*.* **A** *PRRX1* knockdown in different HCC cell lines as determined by qPCR analysis. The knockdown was induced by transfection of 25 nM siPRRX1. Samples were taken after 48 h; bars indicate mean ± SD and are representative of 3 experiments, each in triplicate. **B** *ZEB1* and *ZEB2* expression as determined by qPCR 48 h after siPRRX1 knockdown. Bars indicate mean ± SD and representative of 3 experiments each in triplicates. **C** Cell migration scratch assay 24 h after siPRRX1 transfection. Bars indicate mean ± SD of gap distance (*n* = 18 equidistant measurements) after scratching the monolayer with a 200 μl pipette tip. **D** MTT proliferation assay as measured 48 h after siPRRX1 transfection. Experiment was repeated at least 3 times. Bars indicate mean ± SD, *n* = 12 per group (4 from each experiment). **E** Colony formation assay 8 days after *PRRX1* knockdown. The experiment was repeated at least 3 times. CV = crystal violet. Bars show CV quantification mean values ± SD, *n* = 9 per group from one representative experiment

We knocked down *PRRX1* in HCC cell lines to experimentally validate its relationship with *ZEB1/2* (Fig. 4a). Consistent with the positive correlation with *ZEB1/2* seen in patient datasets, the knockdown of *PRRX1* caused a significant downregulation of *ZEB1* in all three cell lines tested. *ZEB2* was less strongly regulated and changed significantly only in two cell lines (Fig. 4b). Furthermore, we tested the ability of the cells to migrate (as key EMT phenotype). Under basal conditions, the cell line HLF migrated more than HUH7, SNU398 and Hep3B cells. However, cell migratory response was variable upon *PRRX1* knockdown, being increased in HUH7 and Hep3B cells, reduced in HLF and unchanged in SNU398 cells (Fig. 4c). This variability may be linked to the differentiation status of the cells. Further, HCC cell lines showed an increase in cell proliferation after 48 h (Fig. 4d) and clonogenicity after 8 days upon *PRRX1* knockdown (Fig. 4e). Altogether, these findings suggest that modulating *PRRX1* alters *ZEB1/2* and is accompanied by various cellular phenotypes associated with cancer.

### PRRX1 and HCC metabolism

**Fig. 5:**
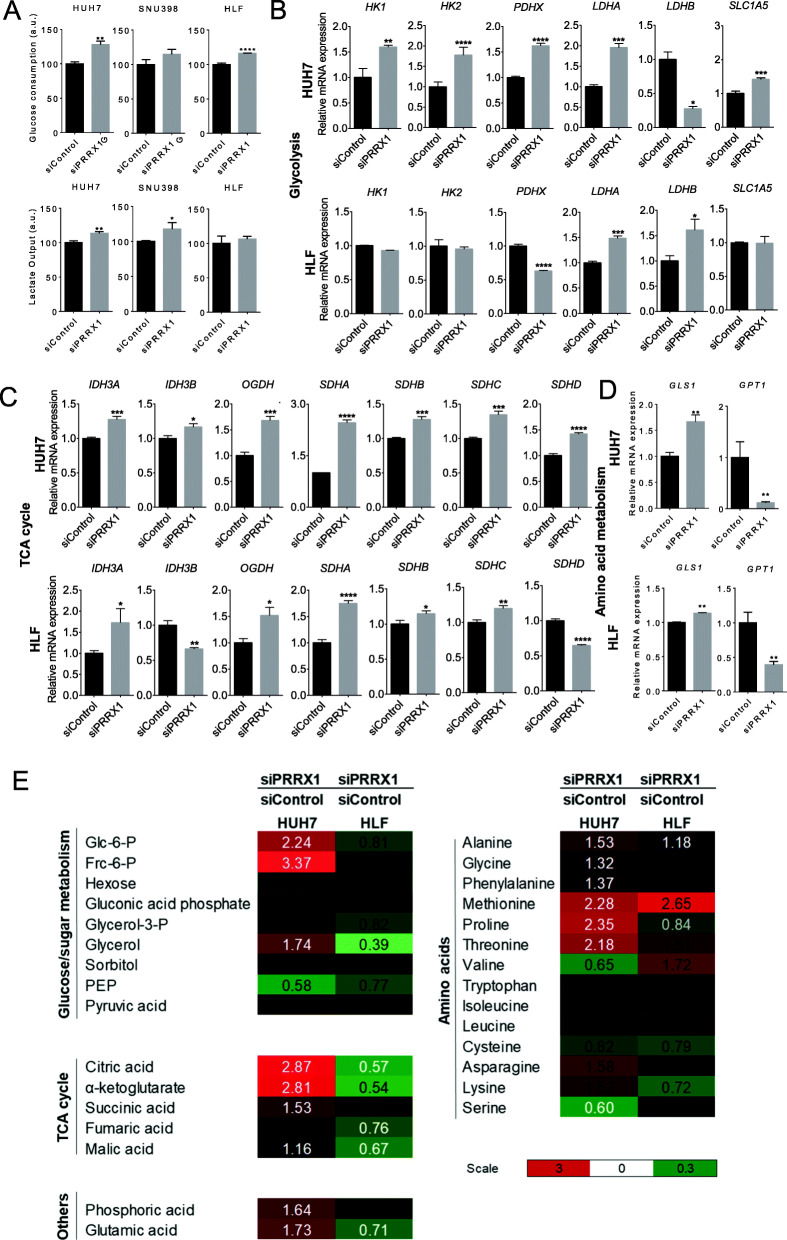
*PRRX1* and metabolism of HCC cells. **A** Glucose consumption and lactate output were measured 48 h after siPRRX1 transfection. Bars indicate mean ± SD and a representative picture of 3 experiments is shown, each in triplicate. **B** Expression of genes involved in glycolysis were determined by qPCR 48 h after siPRRX1 transfection in HUH7 and HLF cells. Bars indicate mean ± SD and representative of 3 experiments each in triplicates. **C** Expression of genes involved in the TCA cycle were analyzed by qPCR 48 h after siPRRX1 transfection in HUH7 and HLF cells. Bars indicate mean ± SD and representative of 3 experiments, each in triplicates. **D** Expression of genes involved in amino acid metabolism as determined by qPCR 48 h after siPRRX1 transfection in HUH7 and HLF cells. Bars indicate mean ± SD and representative of 3 experiments each in triplicates. **E** Alterations of metabolites. Heatmap of changes in metabolite levels of glycolysis, TCA cycle and amino acids in HUH7 and HLF cells 48 h after siPRRX1 transfection. *n* = 3 per cell line and condition. White numbers indicate a *p* < 0.05; black numbers are not significant. The experiment was performed in triplicate

Our previous work revealed consistent metabolic gene changes in HCC [24]. Thus, we further interrogated metabolic alterations, based on the negative correlation between *PRRX1* expression and metabolic genes we observed in the array analyses. To first test a functional link, we measured glucose consumption and lactate output, which are indicative of the Warburg effect (aerobic glycolysis) and are well known to be elevated in cancer. Indeed, the knockdown of *PRRX1* led to an increased glucose consumption in HUH7 and HLF, with a similar upward trend in SNU398 cells (Fig. 5a). In accordance, lactate secretion into the supernatant was increased in HUH7 and SNU398 significantly, and by tendency in HLF cells (Fig. 5a). Based on these findings, distinct gene expression patterns of key components of the glycolytic pathway were determined. A strong effect was monitored in HUH7, with hexokinases 1/2 being increased upon *PRRX1* knockdown, as were *PDHX*, *LDHA*, and *SLC1A5* (Fig. 5b). In contrast, lactate dehydrogenases B (*LDHB)* was reduced. Effects in HLF were less pronounced, but *LDHA/B*, which catalyze the conversion of pyruvate to lactate under anaerobic conditions, were strongly increased (Fig. 5b). Notable alterations of tricarboxylic acid cycle (TCA) genes were also detected in both cell lines. As shown in Fig. 5c, crucial enzymes of the TCA cycle were significantly increased in both cell lines upon *PRRX1* knockdown, e.g. isocitrate dehydrogenases 3A/B (*IDH3A/B*), oxoglutarate dehydrogenase (*OGDH*), several succinate dehydrogenase complex flavoprotein subunits (*SDH*), fumarate hydratase (*FH*), and others (Fig. 5c, Fig. S8A). A few tested targets were reduced upon *PRRX1* knockdown, e.g. phosphoenolpyruvate carboxykinase 1 (*PCK1*), which is responsible for the regulation of gluconeogenesis, and the succinate-CoA ligase GDP/ADP-forming subunit 1 (*SUCLG1*) in both cell lines (Fig. S8A). We also accessed amino acid metabolism genes, especially those involved in glutaminolysis and found them to be deregulated upon *PRRX1* knockdown although for some the direction was cell type dependent (Fig. 5d, Fig. S8B). Considering glutaminase 1 (*GLS1*), which catalyzes the hydrolysis of glutamine to glutamate, the upregulation was consistent in both lines whereas glutamate-pyruvate transaminase 1 (*GPT1*) was strongly repressed (Fig. 5d). Based on the plethora of metabolic gene expression changes upon *PRRX1* modulation, we performed mass spectrometry-based metabolomics to determine changes at the metabolite level. Gas chromatography-mass spectrometry revealed alterations in glycolysis, TCA cycle and amino acid metabolism, with the effect being strongest in HUH7 (the well differentiated cell) (Fig. 5e). While major changes in glycolysis were restricted to the increase of glucose-6-phosphate and fructose-6-phosphate (in HUH7), most analyzed metabolites in TCA cycle were increased. In contrast, HLF cells showed less significant metabolite changes and for all altered analytes, a reduction upon *PRRX1* knockdown was observed. Consistent with these findings, an increase in several amino acids was found (except for serine that was significantly reduced) in HUH7 (Fig. 5e), whereas in HLF, the effects were minor.

**Fig. 6:**
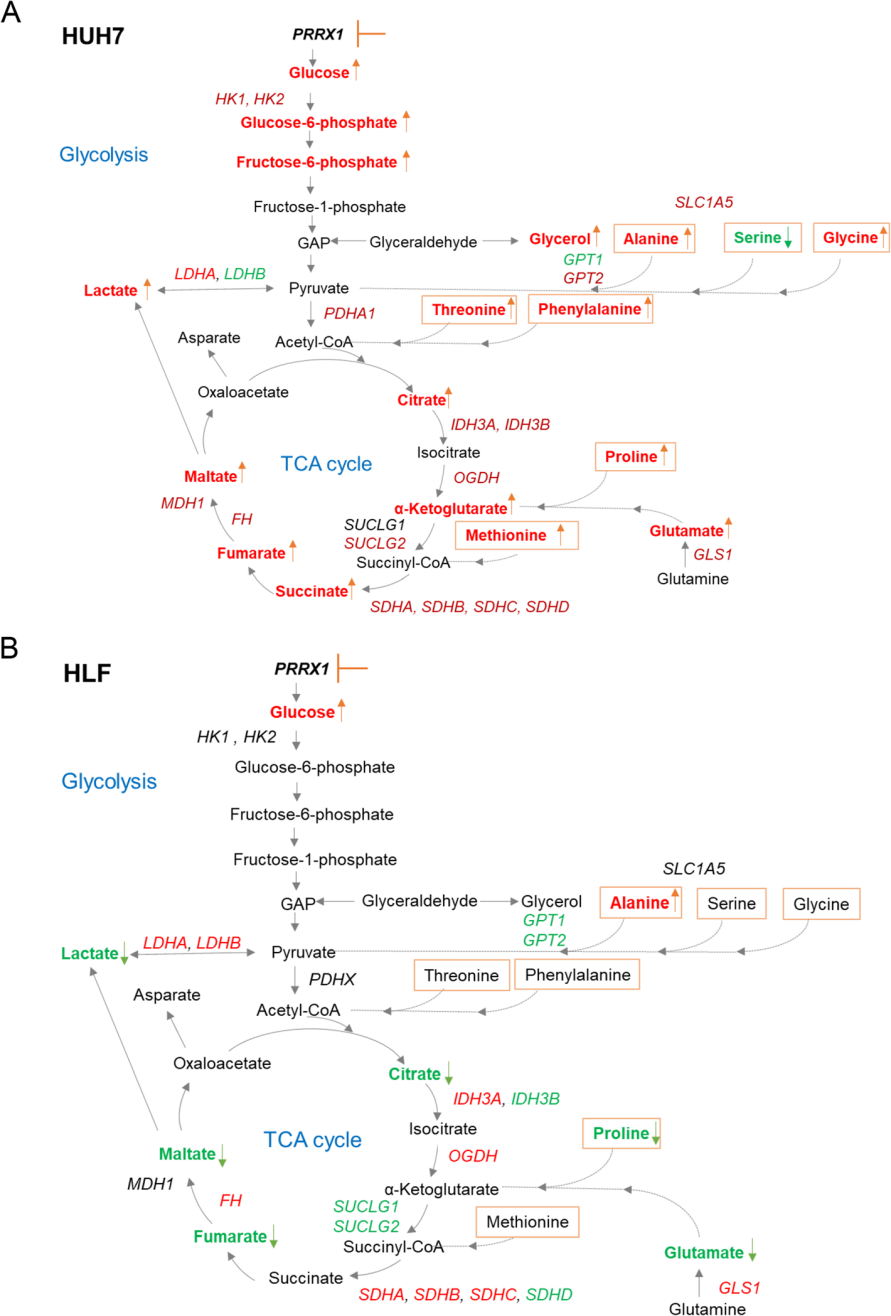
Summary of metabolic changes after knockdown of *PRRX1* (**A**) in HUH7 and (**B**) in HLF cells. Red indicates upregulation, green indicates downregulation of genes or metabolites, *italics:* gene expression, bold: dysregulated metabolites. See text for details

These complex changes on metabolite and gene expression levels are illustrated in Fig. 6a (HUH7) and 6B (HLF) in which affected components of glycolysis and TCA cycle are marked. Taken together, these data support that *PRRX1* controls metabolism in HCC cells in a highly context dependent manner.

## Discussion

Although previous studies investigated the abundance of the transcriptional co-activator *PRRX1* in HCC [12, 13], conflicting expression patterns were reported and *PRRX1* co-regulated genes were not entirely known. In addition, the functions of *PRRX1* in HCC remained largely unknown. In this study, we combined bioinformatics analysis of a large cohort averaging over 1000 HCC tumour samples to resolve the expression pattern of *PRRX1* in HCC. The analysis was complemented with experiments to gain insight on the functional relevance of *PRRX1* in this cancer type. We found that *PRRX1* is frequently upregulated in human HCC cohorts, suggesting that its function is unilateral in HCC. But in our analysis, and consistent with the previous report by Hirata et al. [12]*, PRRX1* level did not correlate with several clinicopathological parameters of HCC patients. Although Hirata et al. found that high *PRRX1* expression predicted longer OS, we observed a similar but marginal tendency that was not statistically significant. We attribute this discrepancy to differences in the number of samples used for the OS analysis. The study by Fan et al. [13] also pointed to a favorable outcome with high *PRRX1* (determined by immunohistochemistry) as low expression correlated with vascular invasion, intrahepatic and distant metastasis as well as advanced tumour stage. Thus, based on the pattern of association with clinical parameters observed by others and us, *PRRX1* can be considered as a tumour suppressor in HCC, at least in the advanced stages of the disease.

*PRRX1* is a typical example of an enigmatic gene that has context-specific functions in HCC. Consistent with this view, a tumour suppressor function seems incompatible with our observation of elevated *PRRX1* in HCC cohorts. Of note, these cohorts also include TCGA data in which Hirata et al. [12] showed that high *PRRX1* expression predicted better OS. Furthermore, the observation that *PRRX1* expression is significantly altered in tumours with mutations in *CTNNB1*, but not *TP53* implies that its expression patterns could substantially vary with additional molecular stratification.

HCC cell lines also provide evidence of variable *PRRX1* level, with a notable low expression in HLF (a high migrating cell line), although not reflecting *PRRX1* correlation to *CTNNB1* (there is an extensive heterogeneity in the mutational landscape of the cell lines – making it hard to attribute even their phenotypic differences to the various mutations [26]). Nevertheless, *PRRX1* level did not clearly enable discrimination of well from poorly differentiated HCC cell lines unlike we and others have shown for other proteins, e.g. caveolin-1, albumin, alpha fetoprotein, SMADs, and WNT signaling targets [23, 27–29].

Evidence from prior studies in colon, breast, and pancreatic cancer support that *PRRX1* has context-dependent functions – i.e. it can have a tumour promoter or suppressor function. Our experimental data with cell lines support a tumour suppressor function for *PRRX1* in HCC. For example, through in silico analysis we predicted that *PRRX1* likely acts by repressing metabolism in HCC. The knockdown of *PRRX1* promoted an aerobic glycolytic phenotype (i.e. increased glucose consumption and lactate output). In a recent genome-wide association study linking type II diabetes sensitivity with gene expression patterns, the authors showed that *PRRX1* is positively linked with insulin sensitivity, supporting the finding that *PRRX1* is related to glucose metabolism [30]. In HUH7 cells, the TCA cycle was also strongly affected at gene and metabolite levels. These observations reflect a tumour suppressive function, even though more studies are required to validate the role of *PRRX1* in cell metabolism and to define whether it is critical for HCC progression.

An increased proliferation rate upon *PRRX1* knockdown also supports a suppressive function. The observation of these phenotypes, even in the cells with comparatively low basal *PRRX1* (i.e. HUH7 and HLF), raises another possibility that certain functions of *PRRX1* are conserved in the different HCC cell lines regardless of basal expression.

In agreement with other studies, we also observed that *PRRX1* function is ambiguous*,* which likely depends on undetermined factors. For instance, Fan et al. showed an increased migration upon *PRRX1* knockdown in HCC cell lines HEPG2 and SMMC7721 [13]. However, when tested across other cell lines in our work, migration was increased in Hep3B and HUH7 cells, unchanged in SNU398, and reduced in HLF cells. Similarly, with regards to the *PRRX1* expression isoforms, most HCC cells expressed more *PRRX1b* isoform than *PRRX1a*. The exception was SNU398, which showed no change in proliferation and migration after *PRRX1* knockdown. Thus, it could be speculated that *PRRX1a*, rather than *PRRX1b* is involved in its phenotypic roles in HCC. Further functional studies will be required to clearly resolve the conditions under which *PRRX1* may exert specific functions in HCC and the exact contribution of the isoform ratio.

Our analysis of human HCC datasets indicated a direct correlation between *PRRX1* and cancer-related processes, e.g. PI3K-Akt signaling pathway, focal adhesion, extracellular matrix, actin cytoskeleton activities and EMT. The exact role of *PRRX1* in controlling the predicted cancer processes needs empirical validation, especially given that the knockdown of *PRRX1* induced pro-cancer activities whereas its high expression in tumours had marginal clinicopathological correlation. *PRRX1* has been described as EMT gene [8, 9, 15] and prior studies link *ZEB1* with unfavorable clinical outcome in HCC [31–33]. Interestingly, we identified EMT genes *ZEB1/2* as specific candidates that together with *PRRX1* impact on clinical OS outcome. We found that *PRRX1* correlated with *ZEB1/2* in three HCC cohorts analyzed. Whereas none of the three genes independently predicted overall survival in the datasets we investigated, high *PRRX1* and either low *ZEB1* or high *ZEB2* expression predicted a better OS.

Finally, an interesting observation is related to patients with *CTNNB1* mutations. In these patients, *PRRX1* expression was significantly lower whereas *ZEB2* was increased. Also, tumours with *TP53* mutations showed significantly reduced *ZEB1* expression. It can be assumed that distinct cancer subtypes, defined by mutation status, will be associated with *PRRX1*/*ZEB1/2* patterns and thus determine disease mechanisms and therewith outcome. Further work will have to shed light on this aspect.

## Conclusion

We have provided evidence of consistent upregulation of *PRRX1* in human HCC. We propose that this gene has anti-tumour functions, at least in advanced tumour stages based on patients’ OS data and our experimental observations. We identify a novel function of *PRRX1* in modulating glycolysis and TCA cycle, and that *PRRX1* correlated with EMT transcription factors *ZEB1/2* (especially with respect to patients’ survival outcome). These findings will enable further in-depth mechanistic studies of the functional relevance of *PRRX1* in human liver cancer, and its crosstalk with *ZEB1/2*.

## Supplementary Information

**Additional file 1: Figure S1.***PRRX1* expression in HCC. **Figure S2.** Analysis of *PRRX1* expression with respect to clinicopathological variables, and in experimental models. **Figure S3.** Cellular components in which are involved genes positively and negatively correlated with *PRRX1* in TCGA liver cancer data. **Figure S4.** KEGG pathway annotation of *PRRX1* correlated genes in various cancers where its alteration frequency is high. **Figure S5.** Annotation of *PRRX1* inversely correlated genes. **Figure S6.** Correlation and survival analyses of *PRRX1* and its co-expressed genes. **Figure S7.** Survival analysis and expression of *ZEB1/2* also with respect to *TP53* and *CTNNB1* mutation. **Figure 8S.** *PRRX1* and metabolic targets. **Table S1.** Human HCC microarrays. **Table S3.** KEGG pathway annotation of genes co-expressed (positively) with *PRRX1.* **Table S4.** KEGG pathway annotation of genes inversely correlated with *PRRX1.* **Table S5.** GO Biological processes for *PRRX1* positively co-expressed genes. **Table S6.** GO Cellular components for *PRRX1* positively co-expressed genes. **Table S7.** GO Biological processes for *PRRX1* inversely correlated genes. **Table S8.** GO Cellular components for *PRRX1* inversely correlated genes. **Table S9.** Metabolic targets in the *PRRX1* co-expressed gene list. **Table S11.** 148 genes as potential candidates that likely cooperate or are coregulated with *PRRX1.* **Table S12.** *ZEB1* expression in combination with *PRRX1* with respect to the clinicopathological variables (n= number of patients). **Table S13.** *ZEB2* expression in combination with *PRRX1* with respect to the clinicopathological variables (n= number of patients).

**Additional file 2: Table S2.** PRRX1 positively and negatively correlated genes in TCGA liver cancer data.

**Additional file 3: Table S10.** Genes differentially expressed in PRRX1-high tumours relative to *PRRX1*-low tumours.

## Data Availability

The publicly available microarray datasets analyzed in this study (GSE25097, GSE64061, GSE55092, GSE36376, GSE39791, GSE62232, GSE57957 and GSE14520) can be accessed at in the NCBI GEO repository (https://www.ncbi.nlm.nih.gov/geo/), while TCGA liver cancer data can be accessed via cBioPortal platform (http://www.cbioportal.org/) and http://cancergenome.nih.gov.

## Abbreviations

CTNNB1: Catenin beta 1
ECM: Extracellular matrix
EMT: Epithelial-to-mesenchymal transition
FH: Fumarate hydratase
GO: Gene Ontology
GOT1/2: Glutamic-oxaloacetic transaminases 1/2
GLS1: Glutaminase 1
GPT1/2: Glutamate-pyruvate transaminases 1/2
GSEA: Gene Set Enrichment Analysis
HCC: Hepatocellular carcinoma
HK1/2: Hexokinase 1/2
IDH3A/B: Isocitrate dehydrogenases 3A/B
LDHA/B: Lactate dehydrogenase A/B
KEGG: Kyoto Encyclopedia of Genes and Genomes
MDH1: Malate dehydrogenase 1
OGDH: Oxoglutarate dehydrogenase
OS: Overall survival
PCK1: Phosphoenolpyruvate carboxykinase 1
PDHX: Pyruvate dehydrogenase complex
PPIA: Peptidylprolyl isomerase
PRRX1: Paired related homeobox 1
qPCR: quantitative polymerase chain reaction
SDHA/B/C/D: Succinate dehydrogenase complex flavoprotein subunits A/B/C/D
SLC1A5: Transmembrane sodium-dependent transporter of amino acid
SUCLG1/2: Succinate-CoA ligase GDP/ADP-forming subunit 1/2
siRNA: small interfering RNA
TCA: Tricarboxylic acid cycle
TCGA: The Cancer Genome Atlas
TP53: Tumor protein P53
VIM: Vimentin
ZEB1/2: Zinc finger E-box-binding homeobox ½
