# Supplementary material for: Dysregulated paired related homeobox 1 impacts on hepatocellular carcinoma phenotypes
Source: BMC Cancer. 2021 Sep 8;21:1006. doi: 10.1186/s12885-021-08637-3 (PMC8424914; doi:10.1186/s12885-021-08637-3)
Supplement: Supplementary file 1 — Additional file 1: Figure S1.PRRX1 expression in HCC. Figure S2. Analysis of PRRX1 expression with respect to clinicopathological variables, and in experimental models. Figure S3. Cellular components in which are involved genes positively and negatively correlated with PRRX1 in TCGA liver cancer data. Figure S4. KEGG pathway annotation of PRRX1 correlated genes in various cancers where its alteration frequency is high. Figure S5. Annotation of PRRX1 inversely correlated genes. Figure S6. Correlation and survival analyses of PRRX1 and its co-expressed genes. Figure S7. Survival analysis and expression of ZEB1/2 also with respect to TP53 and CTNNB1 mutation. Figure 8S. PRRX1 and metabolic targets. Table S1. Human HCC microarrays. Table S3. KEGG pathway annotation of genes co-expressed (positively) with PRRX1. Table S4. KEGG pathway annotation of genes inversely correlated with PRRX1. Table S5. GO Biological processes for PRRX1 positively co-expressed genes. Table S6. GO Cellular components for PRRX1 positively co-expressed genes. Table S7. GO Biological processes for PRRX1 inversely correlated genes. Table S8. GO Cellular components for PRRX1 inversely correlated genes. Table S9. Metabolic targets in the PRRX1 co-expressed gene list. Table S11. 148 genes as potential candidates that likely cooperate or are coregulated with PRRX1. Table S12. ZEB1 expression in combination with PRRX1 with respect to the clinicopathological variables (n= number of patients). Table S13. ZEB2 expression in combination with PRRX1 with respect to the clinicopathological variables (n= number of patients). [file 12885_2021_8637_MOESM1_ESM.pdf]

SUPPLEMENTARY DATA

Dysregulated *Paired related homeobox 1* impacts on hepatocellular carcinoma phenotypes

Weronika Piorońska, Zeribe Chike Nwosu, Mei Han, Michael Büttner, Matthias Philip Ebert, Steven Dooley, Christoph Meyer.

Supplemental Materials & Methods

siRNA transfection

The sequence of siPRRX1 (UUCUGAGUUCAGCUGGUCAUUGUCC), not distinguishing the *PRRX1* isoforms, was obtained from published paper[8], and purchased from Eurofins Genomics (Ebersberg, Germany). Transfection of siRNAs or non-targeting control (siCon) (Qiagen, Hilden, Germany) was performed with Lipofectamine RNAiMAX according to manufacturer's instructions (Invitrogen, Darmstadt, Germany). For the transfection, cells were seeded into 12 well plates (Greiner Bio-One, Frickenhausen, Germany) and allowed to attach overnight (o/n). Next day, medium containing transfection reagent and siRNA mix was given and the cells incubated for 24 h. Thereafter, cells were cultured with fresh complete media for the indicated duration of experiment.

RNA isolation

Total RNA was isolated using InviTrap Spin Universal RNA Mini Kit according to manufacturer's instruction (Stratec Biomedical AG, Germany). RNA concentration was quantified using Infinite 200 NanoQuant Plate (Tecan, Austria). Subsequently, RNA (0.5 or 1 µg) was reverse transcribed to cDNA using SuperScript II First Strand Kit (Invitrogen, USA).

Supplemental Figures

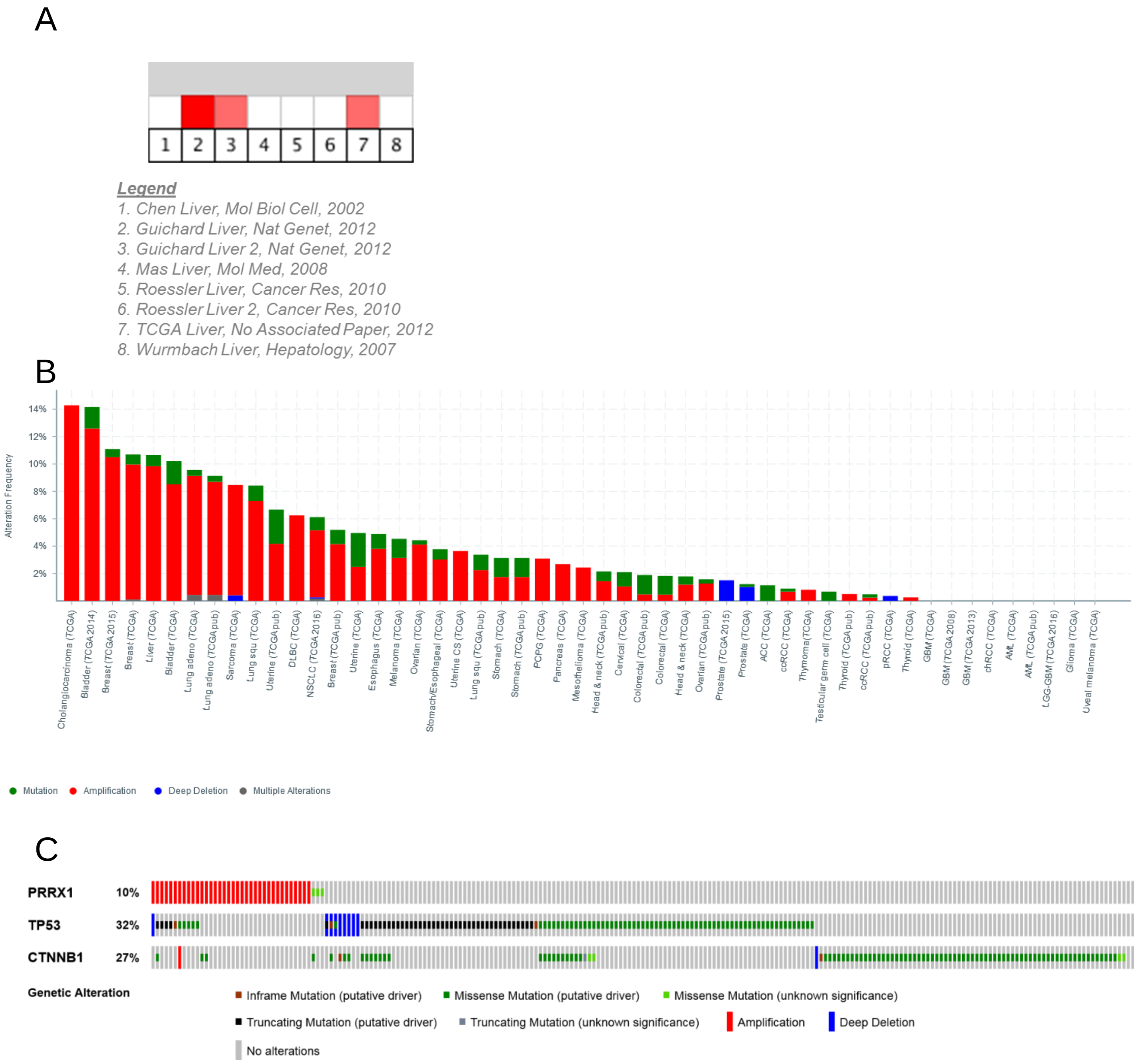

Figure S1. *PRRX1* expression in HCC*.* **(A)** *PRRX1* expression in 8 HCC datasets available in Oncomine. Red - upregulation, white – not significantly altered. Three datasets including The Cancer Genome Atlas (TCGA) liver cancer data (position 7) show *PRRX1* overexpression. **(B)** Analysis of *PRRX1* alteration frequency across TCGA cancer collectives in cBioPortal (http://cbioportal.org/). **(C)** Oncoprint of *PRRX1* along with frequently mutated genes in HCC – *TP53* and *CTNNB1* as visualized in cBioPortal.

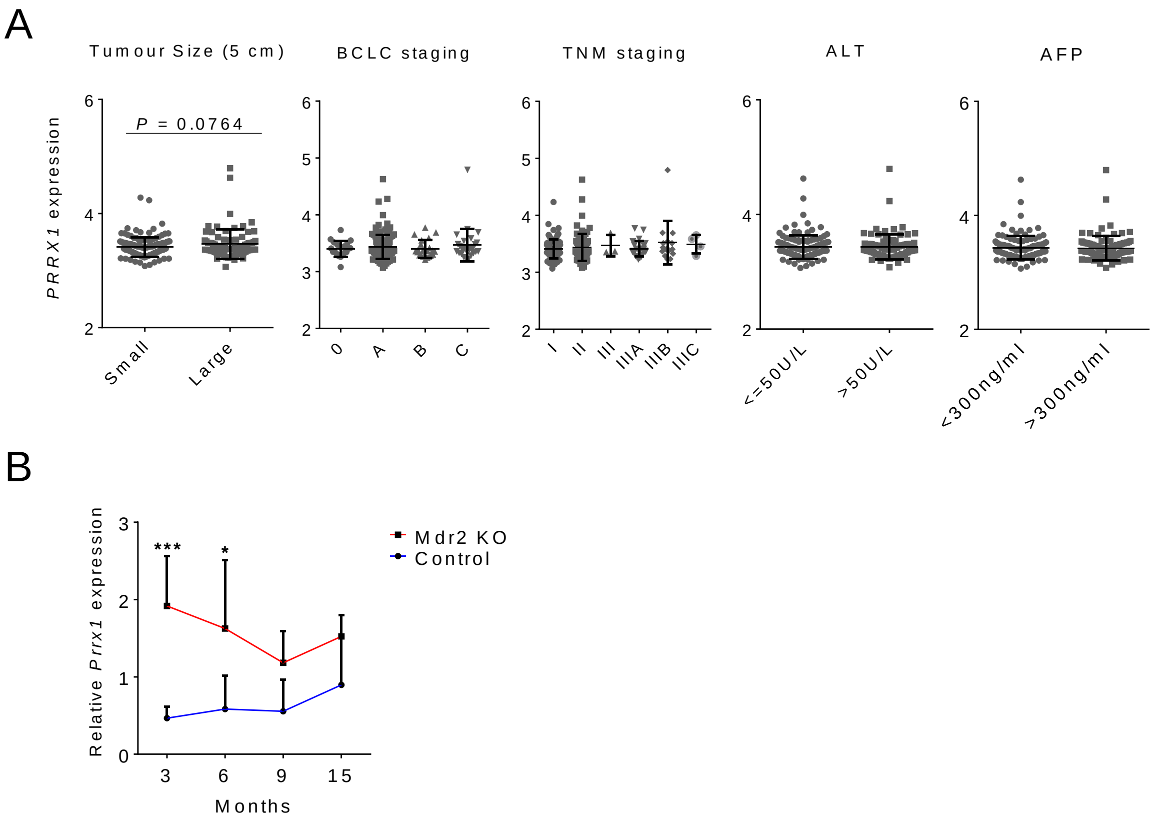

Figure S2. Analysis of *PRRX1* expression with respect to clinicopathological variables, and in experimental models. **(A)** *PRRX1* expression based on tumour size (small n=140, large n=80), tumour staging (TNM: I n=93, II n=77, III n=3, IIIA n=27, IIIB n=15, IIIC n=4; BCLC: 0 n=20, A n=148, B n=22, C n=29), alanine transaminase (ALT <=50U/L n=130, >50U/L n=91) and alpha fetoprotein (AFP <300ng/ml n=118, >300ng/ml n=100) in GSE14520 dataset. **(B)** *Prrx1* expression in control and *Mdr2* KO mice in 3 months (control n=3, *Mdr2* KO n= 4), 6 months (control n=4, *Mdr2* KO n=5), 9 months (control n=4, *Mdr2* KO n=4) and 15 months (control n=5, *Mdr2* KO n=5). Asterisks refer to the difference between control and *Mdr2* KO in in the same time point.

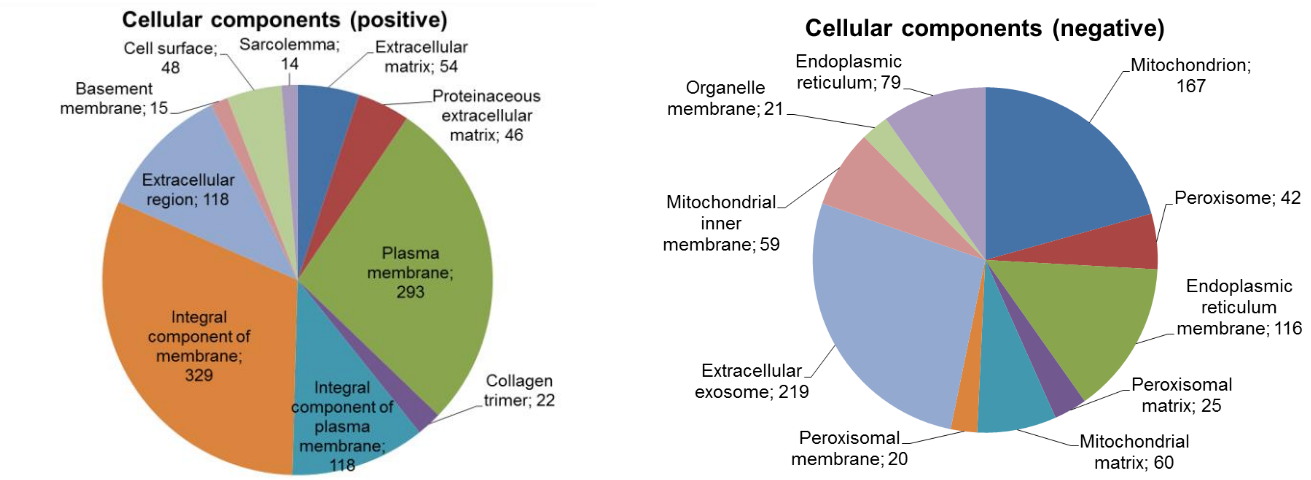

Figure S3. Cellular components in which are involved genes positively and negatively correlated with *PRRX1* in TCGA liver cancer data.

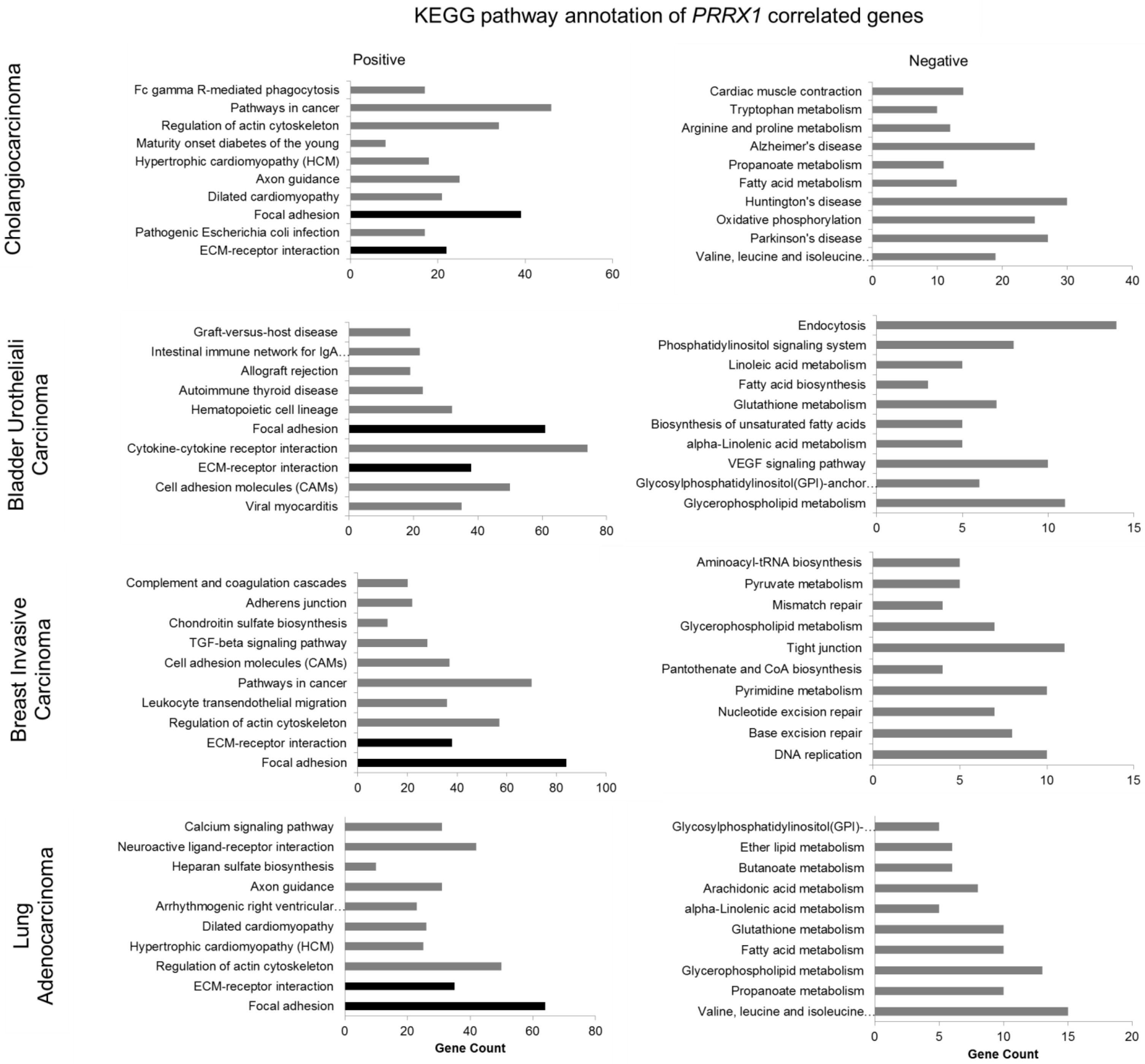

Figure S4. KEGG pathway annotation of *PRRX1* correlated genes in various cancers where its alteration frequency is high. Data were plotted with a gene list obtained from cBioPortal (www.cbioportal.org).

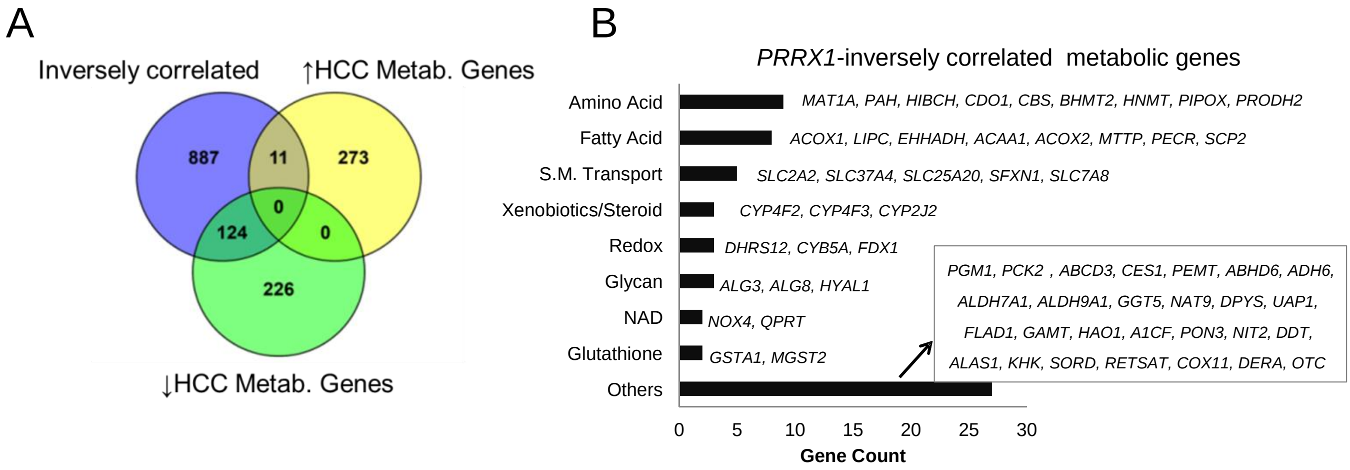

Figure S5. Annotation of *PRRX1* inversely correlated genes. **(A)** Venny diagram for number of genes inversely correlated with *PRRX1* in TCGA liver cancer data overlapped with genes consistently up or downregulated in HCC (Nwosu et al., 2017). **(B)** Metabolic processes reflected by genes correlated with PRRX1 in TCGA and GSE14520 datasets. S.M. – small molecule, NAD – nicotinamide adenine dinucleotide.

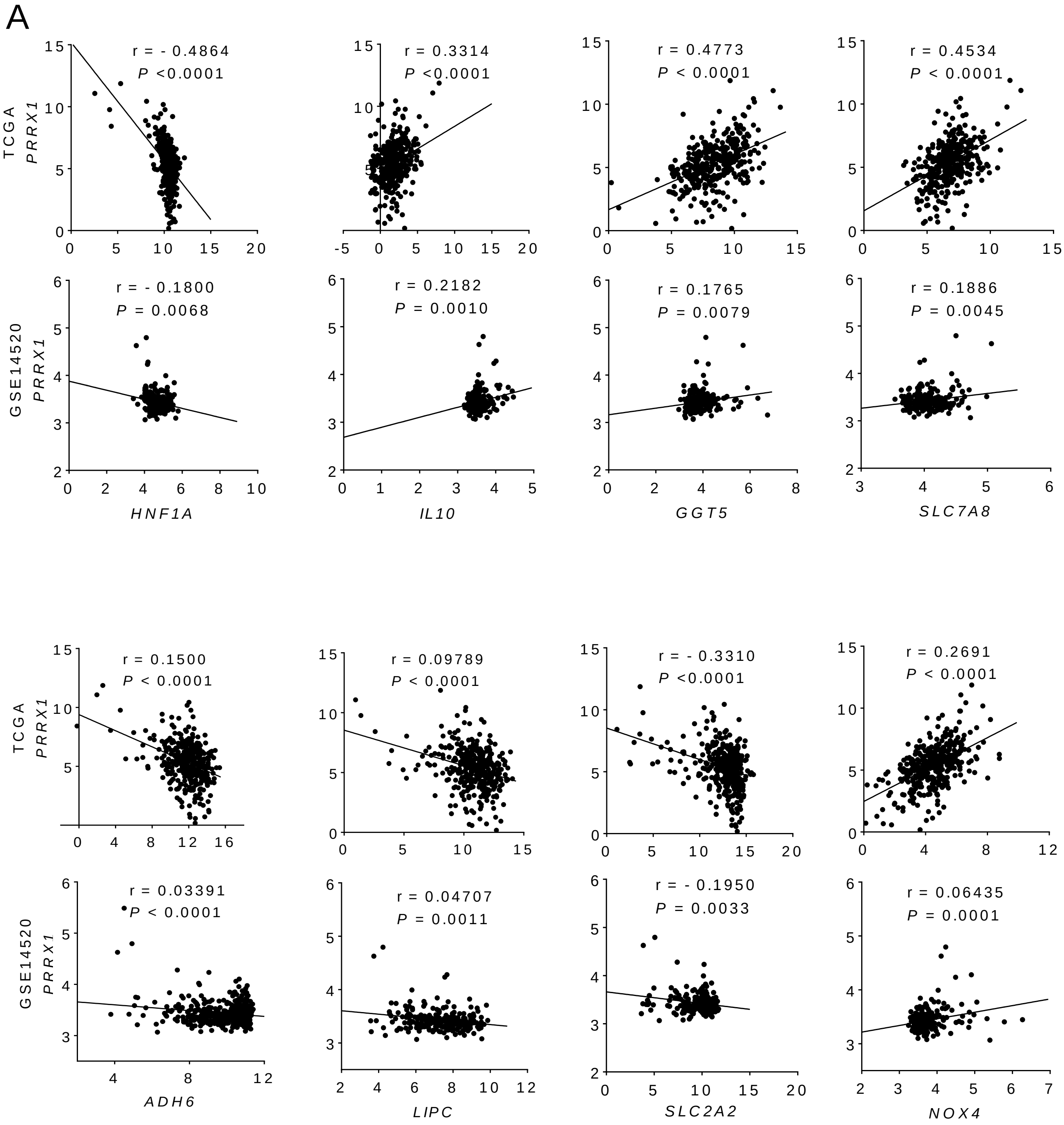
A
TCGA PRRX1
GSE14520 PRRX1
r = - 0.4864
P <0.0001
r = 0.3314
P <0.0001
r = 0.4773
P < 0.0001
r = 0.4534
P < 0.0001
r = - 0.1800
P = 0.0068
r = 0.2182
P = 0.0010
r = 0.1765
P = 0.0079
r = 0.1886
P = 0.0045
HNF1A
IL10
GGT5
SLC7A8
r = 0.1500
P < 0.0001
r = 0.09789
P < 0.0001
r = - 0.3310
P <0.0001
r = 0.2691
P < 0.0001
r = 0.03391
P < 0.0001
r = 0.04707
P = 0.0011
r = - 0.1950
P = 0.0033
r = 0.06435
P = 0.0001
ADH6
LIPC
SLC2A2
NOX4

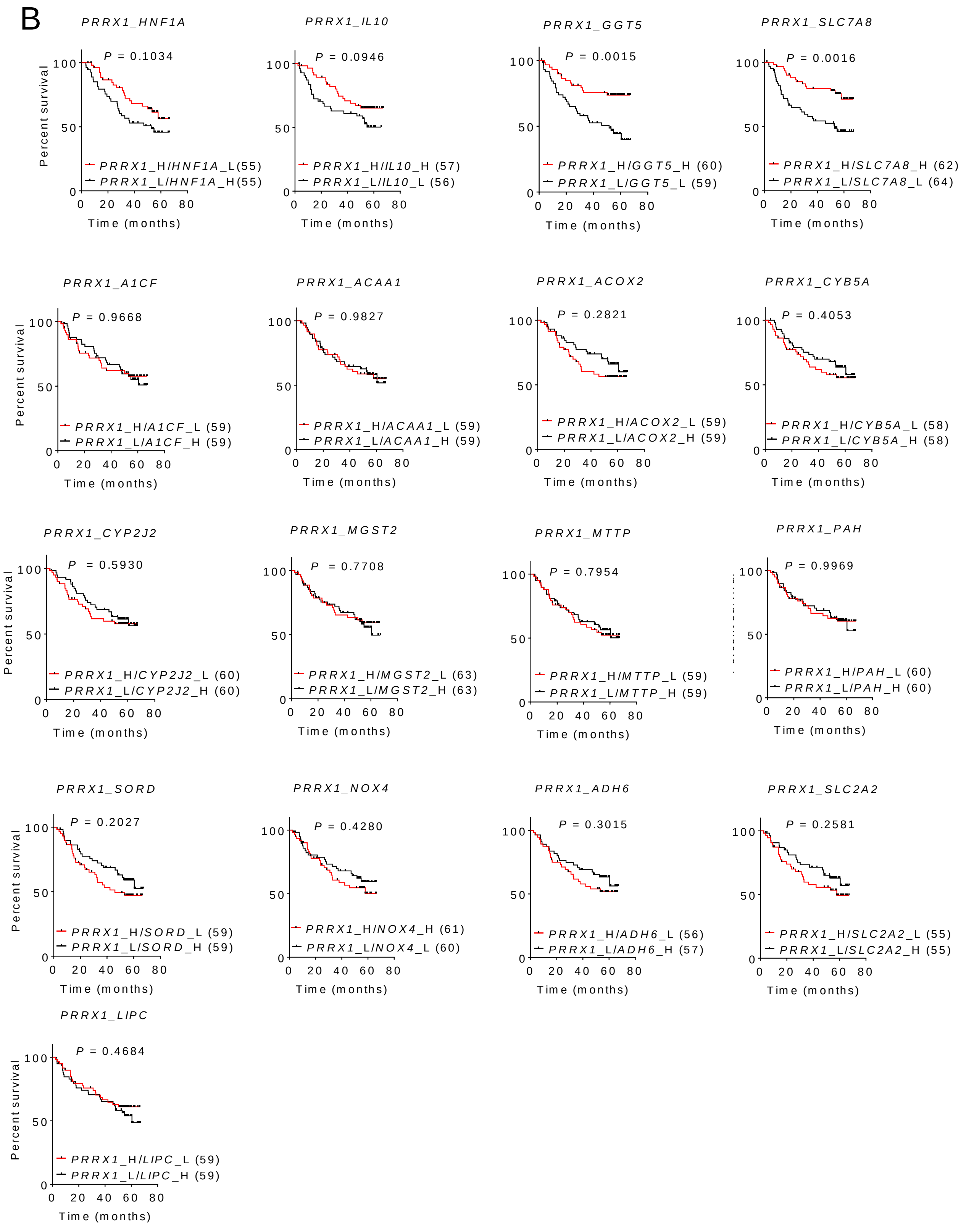

Figure S6. Correlation and survival analyses of *PRRX1* and its co-expressed genes. (A) Pearson correlation analysis of *PRRX1* and *HNF1A*, *IL10*, *GGT5*, *SLC7A8*, *ADH6*, *LIPC*, *SLC2A2*, *NOX4* in TCGA and GSE14520 collectives. **(B)** Kaplan Meier OS analysis (logrank test) of top correlated genes, in combination with *PRRX1* in GSE14520 dataset. Number of patients in brackets. H= high, L= low expression.

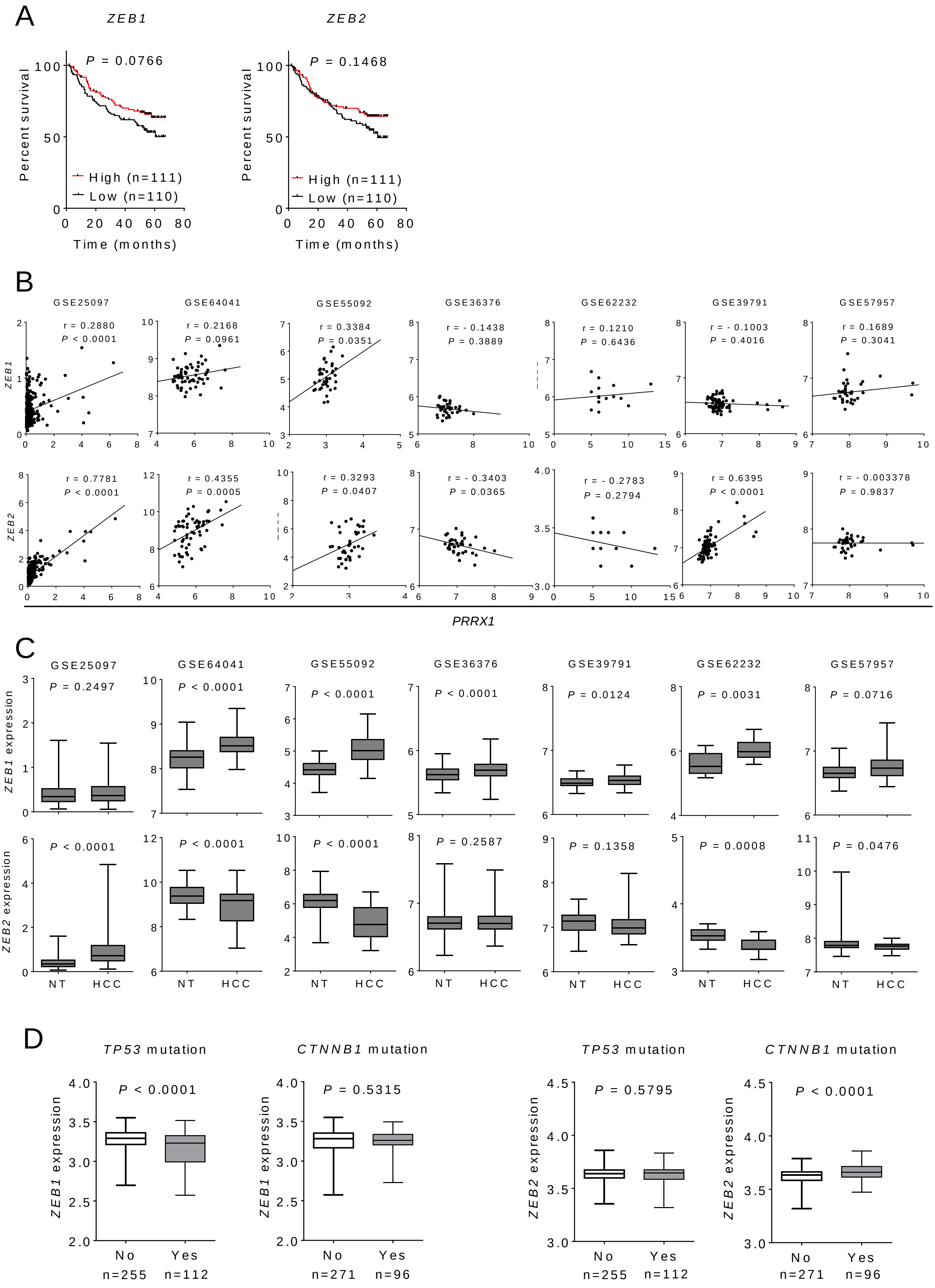
A
ZEB1
P = 0.0766
Percent survival
High (n=111)
Low (n=110)
Time (months)
ZEB2
P = 0.1468
Percent survival
High (n=111)
Low (n=110)
Time (months)
B
GSE25097
GSE64041
GSE55092
GSE36376
GSE62232
GSE39791
GSE57957
ZEB1
r = 0.2880
P < 0.0001
r = 0.2168
P = 0.0961
r = 0.3384
P = 0.0351
r = - 0.1438
P = 0.3889
r = 0.1210
P = 0.6436
r = - 0.1003
P = 0.4016
r = 0.1689
P = 0.3041
ZEB2
r = 0.7781
P < 0.0001
r = 0.4355
P = 0.0005
r = 0.3293
P = 0.0407
r = - 0.3403
P = 0.0365
r = - 0.2783
P = 0.2794
r = 0.6395
P < 0.0001
r = - 0.003378
P = 0.9837
PRRX1
C
GSE25097
GSE64041
GSE55092
GSE36376
GSE39791
GSE62232
GSE57957
ZEB1 expression
P = 0.2497
P < 0.0001
P < 0.0001
P < 0.0001
P = 0.0124
P = 0.0031
P = 0.0716
ZEB2 expression
P < 0.0001
P < 0.0001
P < 0.0001
P = 0.2587
P = 0.1358
P = 0.0008
P = 0.0476
NT
HCC
D
TP53 mutation
P < 0.0001
ZEB1 expression
No
Yes
n=255
n=112
CTNNB1 mutation
P = 0.5315
ZEB1 expression
No
Yes
n=271
n=96
TP53 mutation
P = 0.5795
ZEB2 expression
No
Yes
n=255
n=112
CTNNB1 mutation
P < 0.0001
ZEB2 expression
No
Yes
n=271
n=96

Figure S7. Survival analysis and expression of *ZEB1/2* also with respect to *TP53* and *CTNNB1* mutation. (A) Kaplan Meier OS analysis (logrank test) for patients with high (n=111) and low (n=110) *ZEB1 or ZEB2* expression in GSE14520 dataset. **(B)** Pearson correlation of *PRRX1* and *ZEB1* or *ZEB2* in human HCC colectives. **(C)** *ZEB1* and *ZEB2* expression in human HCC cohorts. Data analysed with Student's t-test. NT= non tumours, HCC= hepatocellular carcinoma. Information about sample size per cohort is contained in Table S1. **(D)** *ZEB1* and *ZEB2* expression in patients with *TP53* or *CTNNB1* mutation in HCC data from TCGA.

A

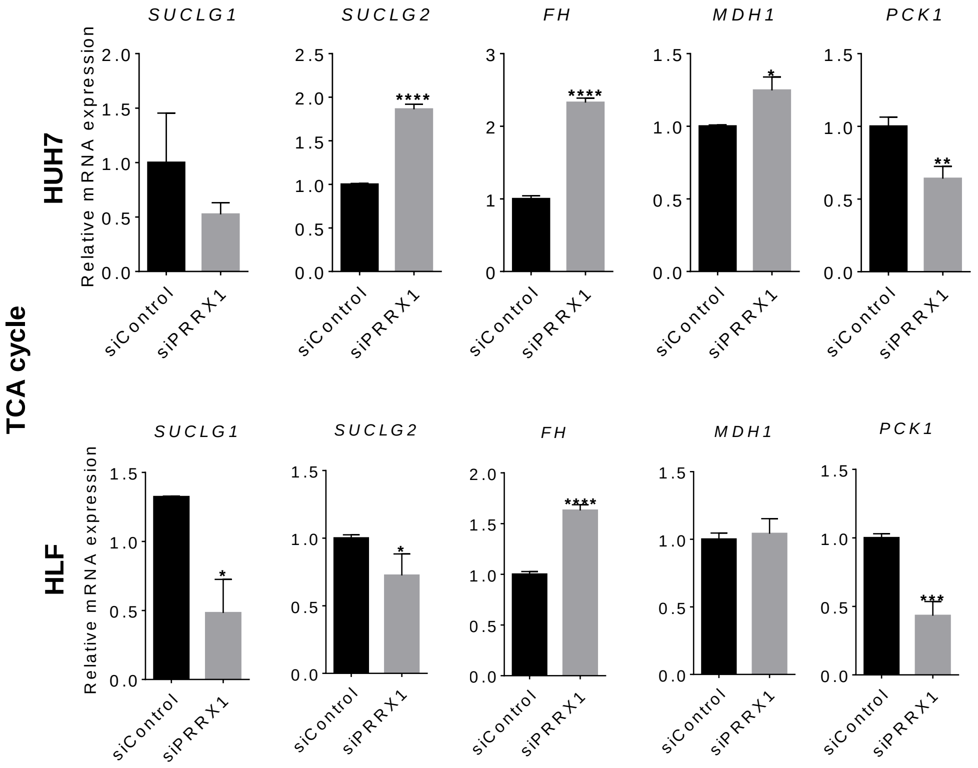
TCA cycle
HUH7
HLF
SUCLG1
SUCLG2
FH
MDH1
PCK1
Relative mRNA expression
siControl
siPRRX1

B

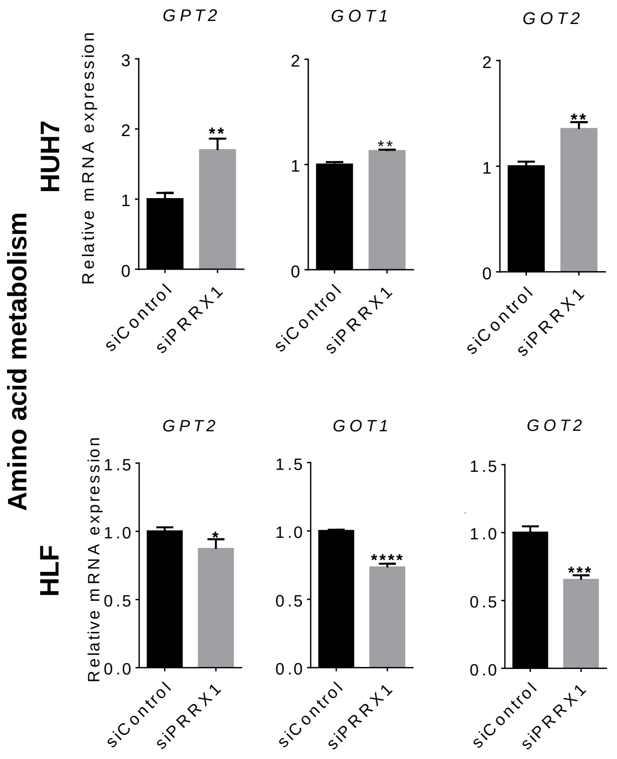
Amino acid metabolism
HUH7
HLF
GPT2
GOT1
GOT2
Relative mRNA expression
siControl
siPRRX1

Figure S8. *PRRX1* and metabolic targets. (A) Expression of genes involved in the TCA cycle as determined by qPCR 48 h after siPRRX1 transfection in HUH7 and HLF cells. Bars indicate mean ± SD and representative of 3 experiments each in triplicates. **(B)** Expression of genes involved in amino acid metabolism as determined by qPCR 48 h after siPRRX1 transfection in HUH7 and HLF cells. Bars indicate mean ± SD and representative of 3 experiments each in triplicates.

Table S1. Human HCC microarrays

	Main etiology	No. of human tissues NL/NT	HCC
TCGA	HBV, HCV & others	[a]47	371
GSE25097	NA	[a]243	268
GSE64041	NA	[a]60	60
GSE55092	HBV	81	39
GSE36376	HBV	[a]32	38
GSE39791	HBV	[a]72	72
GSE62232	HBV, HM	10	17
GSE57957	HBV	[a]39	39
GSE14520	HBV	[NL]220	225
	Total:	**804**	**1129**

[a] - adjacent non-tumorous
NL - normal liver
NT - Non-tumour
NA - etiology is not available
HBV - hepatitis B virus
HCV - hepatitis C virus
HCC - hepatocellular carcinoma

Table S2. *PRRX1* positively and negatively correlated genes in TCGA liver cancer data (attached as a separate Excel file)

Table S3. KEGG pathway annotation of genes co-expressed (positively) with *PRRX1*

KEGG Pathway	Gene Symbols
hsa05200:Pathways in cancer	*ADCY3, FGFR1, ADCY2, ADCY7, FGF9, STAT5A, LPAR4, TGFB3, PML, FGF10, GNG11, LPAR1, MMP2, GLI3, CXCL12, GLI1, EDNRA, PLCB4, RASGRP4, BCL2, PLEKHG5, RALB, PIK3R5, GNG2, PIK3R3, LAMB1, FGF1, CSF1R, PTGER2, PTGER3, RUNX1T1, FZD2, MAPK10, COL4A6, FZD7, RALGDS, LAMA2, LAMA4, LPAR5, LPAR6, NTRK1, PDGFRA, PDGFRB, GNB4, PTCH2, ABL1*
hsa04020:Calcium signaling pathway	*ORAI2, ADCY3, SLC8A3, CCKAR, GNA15, ADCY2, CYSLTR1, ADCY7, LHCGR, ITPKB, EDNRA, HRH1, PLCB4, PDE1B, PDE1A, PTGER3, MYLK2, GRM1, ATP2A3, PLN, HTR7, ATP2A1, CACNA1G, PDGFRA, PDGFRB, CACNA1C, HTR2A*
hsa04510:Focal adhesion	*CAV1, PPP1R12B, TNC, PPP1R12C, COL3A1, ITGA10, VCL, BCL2, COL6A3, COL6A2, COL6A1, PIK3R5, LAMB1, PIK3R3, THBS2, MYLK2, MAPK10, COL5A2, COL4A6, LAMA2, LAMA4, CCND2, FYN, ITGA8, COL1A2, PDGFRA, PDGFRB, COL1A1, PARVA*
hsa04360:Axon guidance	*PLXNA1, EFNB3, LIMK1, DPYSL5, NTNG1, NTNG2, L1CAM, LRRC4C, CXCL12, SLIT2, EPHA3, EPHA5, NCK2, SEMA6B, UNC5B, FYN, SRGAP3, SEMA3A, UNC5C, ABL1*
hsa04974:Protein digestion and absorption	*SLC8A3, ATP1B3, COL13A1, COL3A1, ELN, SLC7A8, COL5A2, COL4A6, SLC1A5, COL14A1, COL6A3, COL6A2, COL1A2, COL12A1, COL6A1, COL1A1*
hsa04151:PI3K-Akt signaling pathway	*FGFR1, FGF9, TNC, COL3A1, LPAR4, FGF10, ITGA10, GNG11, LPAR1, IFNA1, BCL2, COL6A3, COL6A2, COL6A1, GNG2, PIK3R5, PIK3R3, FGF1, LAMB1, THBS2, CSF1R, COL5A2, COL4A6, LAMA2, LAMA4, LPAR5, CCND2, LPAR6, ITGA8, COL1A2, PDGFRA, PDGFRB, JAK2, GNB4, COL1A1*
hsa04512:ECM-receptor interaction	*TNC, COL3A1, ITGA10, COL5A2, COL4A6, LAMA2, LAMA4, ITGA8, COL6A3, COL6A2, COL1A2, COL6A1, COL1A1, LAMB1, THBS2*
hsa04725:Cholinergic synapse	*ADCY3, ADCY2, ADCY7, GNG11, KCNJ12, KCNQ4, CHRM4, PLCB4, FYN, BCL2, GNG2, PIK3R5, GNB4, JAK2, PIK3R3, CACNA1C, CHAT*
hsa04540:Gap junction	*ADCY3, PLCB4, ADCY2, ADCY7, DRD2, PDGFRA, GJA1, PDGFRB, LPAR1, MAPK7, TUBA1A, PRKG1, GRM1, HTR2A*
hsa04611:Platelet activation	*ADCY3, ADCY2, ADCY7, COL3A1, MYLK2, PRKG1, COL5A2, P2RY12, PTGIR, PLA2G4A, PLCB4, FYN, COL1A2, PIK3R5, FCGR2A, COL1A1, PIK3R3*
hsa04810:Regulation of actin cytoskeleton	*ARHGEF4, FGFR1, LIMK1, FGF9, ARHGEF6, PPP1R12B, PPP1R12C, ITGA10, FGF10, PIP5K1C, MYLK2, VCL, INSRR, PFN2, CHRM4, ITGA8, PDGFRA, PDGFRB, PIK3R5, FGF1, PIP4K2A, PIK3R3, SLC9A1*
hsa05146:Amoebiasis	*GNA15, COL3A1, TGFB3, COL5A2, IL10, COL4A6, VCL, LAMA2, LAMA4, PLCB4, COL1A2, PIK3R5, COL1A1, LAMB1, PIK3R3*
hsa04270:Vascular smooth muscle contraction	*ADCY3, KCNMB4, ADCY2, ADCY7, PPP1R12B, PPP1R12C, MRVI1, MYLK2, PRKG1, EDNRA, PRKCQ, PTGIR, PLA2G4A, PLCB4, CACNA1C, PLA2G3*
hsa04750:Inflammatory mediator regulation of TRP channels	*ADCY3, PRKCQ, HRH1, PTGER2, PLA2G4A, PLCB4, ADCY2, ADCY7, ASIC4, NTRK1, PIK3R5, MAPK10, PIK3R3, HTR2A*
hsa04022:cGMP-PKG signaling pathway	*SLC8A3, MEF2C, ADCY3, KCNMB4, ADCY2, ATP1B3, ADCY7, MRVI1, MYLK2, PRKG1, EDNRA, PLCB4, ATP2A3, PLN, ATP2A1, ADRA2A, PIK3R5, PIK3R3, CACNA1C*

Table S4. KEGG pathway annotation of genes inversely correlated with *PRRX1*

KEGG pathway	Gene Symbols
hsa01100:Metabolic pathways	*ALAD, BTD, HMGCR, EHHADH, PPCS, PI4K2B, AGXT, HIBADH, CMBL, FAH, SCLY, MAT1A, ST3GAL6, RGN, DAO, HMGCL, ACSM2B, CRLS1, GATM, FAXDC2, ACSM2A, PDXP, MOGS, CDO1, CHPT1, PNPLA3, HYKK, PGM1, ABAT, FLAD1, PCCB, ACAA1, MPST, ACADSB, AHCY, NAGS, GNE, CYP51A1, ALDOC, CERS4, PAH, AGMAT, ACAT2, GALM, CERS2, IVD, GMPPA, PEMT, IDH1, GALE, MOCS1, FH, MGAT4B, NADK2, ST6GAL1, UAP1, MSMO1, ACY1, B4GAT1, CYP2C9, UPB1, GUSB, MAOA, MAOB, GALT, EPHX2, FDPS, MMAB, TST, POLD4, ATP6V0E2, ALDH2, PON1, PRODH2, QPRT, CYP8B1, DCXR, PON3, ALG14, ACOX2, TM7SF2, ACOX1, SEPHS2, COX11, CYP2J2, AMT, ALG3, ALG5, PPOX, ALG6, ALG8, PMVK, ALG9, ACOT4, AFMID, ALAS1, CRYL1, PIGM, AKR1C4, MCEE, PCYT2, SARDH, AGPAT3, DHCR24, COX15, HYAL1, DDC, ALDH5A1, OTC, PIGV, NDUFC2, GRHPR, PIGP, COQ5, NAPRT, HAO1, ALDH7A1, DHRS3, UMPS, DHRS4, DGAT2, CYP27A1, PANK1, PKLR, UGT2B10, GPAM, ALDH9A1, SLC27A5, XYLB, SORD, HSD3B7, ADH5, NFS1, DHRS4L2, ADH6, ECHS1, DHRS4L1, DPYS, ALDH3A2, PIPOX, UGT1A6, GALK1, MTHFS, CBR1, AKR1A1, DHCR7, PLA2G12B, HAAO, HSD17B6, DMGDH, PNPO, ETNK2, HSD17B4, BDH1, MTMR4, HSD17B7, ACSL5, GBA, NSDHL, HSD17B8, MOGAT3, SHMT1, CHDH, EBP, CES1, FTCD, POLR3GL, PCK2, IDNK, OXSM, HYI, KHK, ADI1, CYP4A11, MPI, SDHC, UCKL1, GAMT, CYP4F3, CYP4F2, HIBCH, LIPC, SCP2, ACSM5, CBS*
hsa04146:Peroxisome	*ACOX2, ACOX1, HACL1, ECH1, EHHADH, PEX6, PEX5, DHRS4L1, PMVK, AGXT, PEX11G, PIPOX, PEX7, PECR, PEX1, PEX19, MPV17L, GSTK1, PEX16, PXMP4, GNPAT, PXMP2, ABCD3, IDH1, DAO, CAT, HSD17B4, HMGCL, ACSL5, ECI2, PAOX, NUDT12, EPHX2, DECR2, CRAT, SOD1, PHYH, HAO1, PEX11A, DHRS4, SCP2, ACAA1*
hsa01130:Biosynthesis ofantibiotics	*TM7SF2, CYP51A1, HMGCR, ALDOC, AMT, EHHADH, ADH5, ECHS1, ACAT2, AGXT, ALDH3A2, CMBL, GALM, AKR1A1, RGN, IDH1, DAO, CAT, HSD17B7, FH, NSDHL, SHMT1, UAP1, MSMO1, ACY1, FAXDC2, OTC, FDPS, PCK2, IDNK, HAO1, ALDH7A1, SDHC, PKLR, PGM1, ALDH2, PCCB, ALDH9A1, CBS, ACAA1*
hsa00260:Glycine, serine and threonine metabolism	*SHMT1, CHDH, GATM, AMT, MAOA, MAOB, GRHPR, AGXT, PIPOX, ALAS1, ALDH7A1, DMGDH, DAO, GAMT, SARDH, CBS*
hsa00071:Fatty aciddegradation	*ECI1, ECI2, ACOX1, ACADSB, EHHADH, ADH5, ECHS1, ADH6, ACAT2, ALDH3A2, CYP4A11, ALDH7A1, ALDH2, ALDH9A1, ACAA1, ACSL5*

Table S5. GO Biological processes for *PRRX1* positively co-expressed genes

GO Biological processes	Gene Symbols
GO:0007155~cell adhesion	*PCDHA2, NUAK1, MYBPC1, IGFBP7, L1CAM, POSTN, DDR2, CXCL12, VCL, WISP1, SRPX, FAP, COL12A1, LOXL2, BOC, ADGRE1, CLCA2, PCDHB6, MFGE8, SSPN, CERCAM, NCAM1, CD33, LSAMP, SIGLEC7, SUSD5, VCAN, CNTN4, TGFB1I1, COL1A1, MFAP4, ADAM12, PARVA, SCN1B, TNC, ITGA10, PCDHGC3, ISLR, IGSF11, COL6A3, COL6A2, COL6A1, COL8A1, LAMB1, ENTPD1, GPNMB, THBS2, HAPLN1, HAPLN3, ADAM23, PCDH10, NID2, EMILIN2, TPBG, COL4A6, PCDH18, EPHA3, EMILIN1, LAMA2, LYVE1, LAMA4, STAB1, ITGA8, ADAM22, FEZ1, CDH11*
GO:0030198~extracellular matrix organization	*LUM, TNC, COL3A1, ELN, ITGA10, POSTN, VIT, DDR2, ABI3BP, FOXF1, FOXF2, COL6A3, COL6A2, COL6A1, COL8A1, LAMB1, COL8A2, LOXL1, HAPLN1, COL13A1, OLFML2B, CCDC80, NID2, SPARC, COL5A2, COL4A6, NDNF, EMILIN1, LAMA2, LAMA4, COL14A1, ITGA8, COL1A2, VCAN, MFAP2, COL1A1, JAM2, MFAP5, JAM3*
GO:0030574~collagen catabolic process	*ADAMTS14, COL13A1, COL3A1, MRC2, COL25A1, MMP14, MMP13, COL5A2, MMP2, COL4A6, MMP11, CTSK, COL6A3, COL1A2, COL6A2, COL12A1, COL6A1, COL1A1, COL8A1, COL8A2*
GO:0007399~nervous system development	*CER1, MEF2C, PCDHA2, GPM6B, ZEB2, L1CAM, MYLIP, ST8SIA2, PAX3, DPF1, SCRG1, GPSM1, DLG4, PTN, SH2B2, DCLK1, EFNB3, SCN2B, LIMK1, MAFB, PCDHB6, MAP1B, DPYSL5, GAS7, PCDH18, SHOX2, NBL1, SLC4A10, CHRDL1, DOK5, LSAMP, ST8SIA4, TMOD2, CNTN4, NRGN, FABP7, SMARCA2, GFRA2, FEZ1, VLDLR*
GO:0007165~signal transduction	*ADCY3, IL19, CRABP2, PLPPR4, STOML3, RCVRN, ITPKB, RADIL, PRKG1, CXCL12, IL17RD, VIPR2, MAP3K6, ANK1, UNC5B, ANK2, ANK3, PPP1R1B, RALB, UNC5C, CHRNA1, MAGI2, PDPN, GEM, CD33, PDGFRB, MAPK7, EXT2, ELK3, ADRA2A, CDC42EP3, CSF1R, ABR, MRC2, TRIM63, LYVE1, CXCL14, ANTXR1, SYNGAP1, PLAU, ZNF536, FGF9, GJA1, DDR2, EDNRA, ARHGAP22, NOD1, PRMT2, WISP1, EVI2A, ARHGAP1, DLG4, MICAL1, FGF1, CHRFAM7A, GPR173, RSU1, LIMK1, ARHGAP28, ARHGAP24, NLRP3, ARHGAP23, GRP, ARHGAP31, DOK1, NCK2, CHRM4, RIN2, SRGAP3, GNB4, INPP4B, NRGN, INPP4A, PPP1R12B, SLC39A12, GNG11, CALCB, PDE1B, BCL11A, LANCL3, PDE1A, PLEKHG5, SH2B2, PLA2R1, ANXA1, DPYSL5, AXL, SPARC, MAPK10, RALGDS, SH3BP5, LSP1, SYDE1, RPS6KA2, TENM3, CHN1, JAK2, PTCH2, IGFBP5, FAM126A, VLDLR*
GO:0030199~collagen fibril organization	*COL14A1, ADAMTS14, SFRP2, LUM, COL3A1, COL1A2, COL12A1, COL1A1, LOXL2, SERPINH1, COL5A2, DDR2, MMP11*
GO:0007411~axon guidance	*SCN1B, L1CAM, GLI3, CXCL12, ANK3, SEMA3A, UNC5C, BOC, FOXD1, GPC1, CSF1R, EFNB3, KIF5A, DRAXIN, DPYSL5, SLIT2, FEZF1, NCAM1, EPHA5, LAMA2, PRKCQ, FYN, NTRK1, CNTN4, BMP7, FEZ1*
GO:0010811~positive regulation of cell-substrate adhesion	*RSU1, FBLN2, FOXF1, CCDC80, PTN, HACD1, JAK2, COL8A1, VIT, ABI3BP, NDNF, EMILIN1*
GO:0001503~ossification	*TWSG1, MGP, GPM6B, LRRC17, SPARC, OSTN, COL5A2, DDR2, CHRDL1, BCL2, RASSF2, TMEM119, BMP7, EXT2, BMP5, TWIST1, CDH11*
GO:0001764~neuron migration	*MEF2C, CCKAR, FGFR1, CDK5R2, AXL, GJA1, PRKG1, CXCL12, NKX6-1, NDNF, MARK1, FEZF1, NAV1, FYN, NTRK2, SEMA3A, MKL1, DCLK1, TWIST1*

Table S6. GO Cellular components for *PRRX1* positively co-expressed genes

GO Biological processes	Gene Symbols
GO:0031012~extracellular matrix	*LTBP2, IGFBP7, TGFB3, FGF10, POSTN, MMP2, NOV, PKM, MMP21, COL12A1, LOXL2, LOXL1, ZP1, CILP, MGP, MFGE8, MMP14, MMP13, NDNF, MMP11, COL1A2, VCAN, TGFB1I1, COL1A1, MFAP4, LUM, TNC, VIM, COL3A1, TIMP2, ABI3BP, COL6A3, COL6A2, COL6A1, ADAMTS12, LAMB1, COL8A1, THBS2, COL8A2, PLAT, HAPLN1, NES, EFEMP1, NID2, EMILIN2, COL5A2, EMILIN1, LAMA2, LAMA4, COL14A1, SFRP1, SFRP2, FBLN2, BMP7*
GO:0005578~proteinaceous extracellular matrix	*PODNL1, LTBP2, MAMDC2, KERA, ADAMTS14, LUM, ELN, POSTN, VIT, TIMP2, MMP2, CPZ, NOV, GPC2, WISP1, EMID1, COL6A3, COL6A2, ADAMTS12, FGF1, GPC1, COL8A2, HAPLN1, ZP1, HAPLN3, PTPRZ1, CRTAC1, OLFML2B, EFEMP1, CILP, MGP, SPARC, EMILIN2, MMP13, COL5A2, SLIT2, NDNF, COL4A6, MMP11, EMILIN1, ADAMTS7, COL14A1, SFRP1, FBLN2, COL1A2, VCAN*
GO:0005886~plasma membrane	*SLC9A9, ADCY3, CADM3, ADCY2, ATP1B3, ADCY7, SLC9A5, SCN3A, LHCGR, SNCA, LPAR4, PLPPR4, L1CAM, SYT6, STOML3, MYLIP, LPAR1, PRKG1, TRAK2, FAP, DYNC2H1, RAB23, ABRA, GNG2, CHRNA1, HCAR1, ADGRB2, CRTAM, CLCA2, CAPNS2, MAGI2, EFNB3, PDPN, COLEC12, PDCD1LG2, MARK1, LPAR5, GPBAR1, LPAR6, HTR7, CD33, PDGFRA, PDGFRB, PMP22, ADD2, KCNMB4, CDK5R2, SCN1B, ARHGEF25, TMX3, ITGA10, CDC42EP3, CDC42EP5, TRPC4, MLC1, ABR, TRPC7, PMEL, PCDH10, GAS1, PCDH19, EPHA3, PCDH18, EPHA5, GGT5, ADAP2, SFRP1, OR6B3, NTRK1, HEPH, CLEC7A, TMEM119, PLAU, SLC9A1, SLC27A1, STEAP4, GYPC, CCKAR, JPH3, LIMA1, KCNAB3, MSR1, GYPE, GRIP1, GPR82, GJA1, KCNJ12, KCNIP4, PKM, EDNRA, NMUR1, NMUR2, CHRFAM7A, FAM129A, KCNG1, CD200R1, GPR173, PCDHB9, FMNL3, PCDHB7, STX1A, ZP1, CLMP, PCDHB6, COL25A1, MMP14, GRM1, OR2W3, SLIT2, SLC26A4, EPB41L3, SGCG, CLIC4, PLXDC1, CYBRD1, SH3KBP1, GRIP2, GNB4, ADAM12, PLA2G3, ARL4C, MAP3K12, PARVA, GPR63, GPM6B, GNG11, CLDN11, ITM2C, PCDHB10, DCHS1, GJC1, PLCL1, IGSF11, KIRREL, CLEC2B, IL10RA, PLEKHG5, LANCL3, SLC4A8, ETV5, AXL, FZD2, MAPK10, FZD7, P2RY12, LSP1, P2RY13, FYN, STAB1, BNC2, CDH19, PLSCR3, MC4R, FCGR2A, ABCC5, GFRA2, HTR2A, CDH11, FAM126A, VLDLR, QRFPR, SLC8A3, GNA15, PLXNA1, GABRB2, OR2J3, SLC7A8, TGFB3, FGF10, MMP2, VIPR2, IL31RA, VCL, PTGIR, ANK1, ANK2, UNC5B, ANK3, RALB, SLCO1C1, UNC5C, MCOLN2, NEGR1, KCND1, MRGPRF, CERCAM, RPH3A, NCAM1, GPR55, LSAMP, EMP3, EMP1, FGFR1, PAM, C3AR1, CAV1, CYSLTR1, DRD2, STK10, KCNA1, PEAK1, NKAIN3, TTYH3, KCNS2, CRB2, RHOBTB2, ADRA2A, ENTPD1, HCN4, PLXND1, CSF1R, AP2M1, KCNB2, ADAM23, SYT11, RAB33A, ABCB5, SLC4A10, LYVE1, SLC7A3, DIO3, ITGA8, CACNA1G, ADAM22, ANTXR1, CPNE2, PIP4K2A, CACNA1C, PLEKHA2, RHOJ, LRRC8B, LRRC8C, UCHL1, RHOQ, DDR2, SDC3, SLC1A5, PCDHGB1, KCNQ4, TMEM173, GPC2, RAET1G, PRMT8, RASL10A, DLG4, ANO4, GPC1,*

	BOC, ATP8B4, EFR3B, PTGER2, PTGER3, RIMBP2, LDLRAD3, PRKCQ, SIGLEC1, CHRM4, CD80, ULBP3, SIGLEC7, CNTN4, JAM2, PRNP, JAM3, PTCHD1, PPP1R12B, VIM, CYTH3, PCDHGC3, RGMA, HRH1, STK32A, MARVELD1, RASGRP4, GPSM1, SH2B2, PIK3R5, PLA2R1, GPNMB, SCN5A, EHD2, MYOF, RNF144A, COL13A1, MAP1B, ANXA1, SPARC, SLC17A7, PPP1R9B, RGS20, SULF2, SLC6A6, FEZ1
GO:0005581~collagen trimer	*MSR1, COL13A1, COL3A1, COL25A1, COLEC12, EMILIN2, SERPINH1, MMP13, COL5A2, COL4A6, EMILIN1, C1QTNF7, COL14A1, EMID1, COL6A3, C1QTNF2, COL1A2, COL6A2, COL12A1, COL6A1, COL1A1, COL8A2*
GO:0005887~integral component of plasma membrane	*SLC8A3, ADCY3, QRFPR, CALHM2, CADM3, ADCY2, PLXNA1, GABRB2, LHCGR, PLPPR4, SLC7A8, LPAR4, LPAR1, SLC26A10, VIPR2, IL17RD, PTGIR, SLCO1C1, CLCA2, CRTAM, EFNB3, PDPN, MRGPRF, SSPN, GPR55, LPAR6, HTR7, CD33, PDGFRA, C3AR1, FGFR1, KCNMB4, CAV1, CYSLTR1, DRD2, KCNA1, XG, ADRA2A, PLXND1, HCN4, ENTPD1, CSF1R, TRPC4, ADAM23, SYT11, PMEL, TRPC7, MRC2, ATP13A2, TPBG, ABCB5, EPHA3, EPHA5, LYVE1, SLC4A10, SEMA6B, GPR34, SLC7A3, ATP2A3, NTRK1, ATP2A1, NTRK2, SLC9A1, CCKAR, STEAP4, GYPC, GYPE, MSR1, PCDHA2, LRRC8C, GJA1, KCNJ12, DDR2, INSRR, TSPAN11, EDNRA, SLC1A5, TSPAN10, NMUR1, GPC1, BOC, MLANA, PTGER2, ADGRE1, PTGER3, PCDHB6, COL25A1, MMP14, GRM1, SLC26A4, CLEC1A, CHRM4, SIGLEC7, JAM2, SLC39A12, GPR1, HRH1, CLEC2B, TSPAN32, SLC4A8, GPNMB, PLA2R1, DCLK1, FAM26E, PTPRZ1, ASIC4, AXL, NLGN2, TSPAN18, P2RY12, SAMD8, P2RY13, STAB1, SLC6A6, TENM3, YKT6, ABCC5, HTR2A*
GO:0016021~integral component of membrane	*SLC9A9, ADCY3, ADCY2, ADCY7, SLC9A5, SCN3A, GBGT1, LHCGR, LPAR4, L1CAM, SYT6, STOML3, LEMD1, LPAR1, SLC26A10, ATCAY, FAM171B, GAB3, FAP, CHRNA1, HCAR1, ADGRB2, RARG, VANGL1, PDPN, PLD5, TMEM132B, TMEM200A, TMEM200C, COLEC12, LHFP, PDCD1LG2, LHFPL2, C14ORF37, LPAR5, GPBAR1, LILRB5, LCTL, LPAR6, CD33, ASPHD2, VSIG8, PDGFRB, TMEM184B, PMP22, EXT2, ORAI2, MGAT5B, TMX3, SFXN3, RIC1, SYNDIG1L, SORCS2, KIAA1549L, TMEM229A, DAPL1, PFN2, XG, FAM162B, SASH1, ACER3, OSBPL5, TRPC4, FAXC, MLC1, OSBPL8, TRPC7, PMEL, PCDH10, CCDC80, GAS1, ATP13A2, PCDH19, PORCN, EPHA3, PCDH18, EPHA5, GGT5, SEMA6B, SFRP1, OR6B3, ATP2A3, APCDD1L, SFRP2, ATP2A1, NTRK2, HEPH, CLEC7A, TMEM119, TLL1, SLC9A1, SLC27A1, STEAP4, JPH3, KCNAB3, MSR1, GYPE, GPR161, GPR82, LTC4S, LRRC15, RNF182, GJA3, DNAJC18, ST6GALNAC3, TMEM108, NMUR1, XYLT1, NMUR2, CHST12, SNPH, GPX8, CHRFAM7A, CD200R1, IL13RA2, PCDHB9, GPR173, PCDHB7, KIAA1324L, STX1A, ZP1, CLMP, PCDHB6, COL25A1, RNF175, OSTM1, MMP14, GRM1, MMP13, OR2W3, SLC26A4, CLEC1A, CHSY3, SGCG, PLXDC1, PLXDC2, CYBRD1, SH3KBP1, SUSD5, CHSY1, ADAM12, SGCA, FUT8, GPR63, GPM6B, CLDN11, ITM2C, PCDHB10, DCHS1, SEC14L1, GJC1, IGSF11, LRRTM3, KIRREL, FMO2, BCL2, IL10RA, ARMCX2, POPDC2, ARMCX1, ABCA13, ARMCX6, KCNE4, CMTM1, A4GALT, PTPRZ1, LRRN3, XKR5, MXRA7, AXL, KIAA1644, MXRA8, FZD2, FZD7, SAMD8,*

	P2RY12, P2RY13, FAM180B, STAB1, PLN, TENM3, CDH19, PLSCR3, KREMEN1, TMTC1, MC4R, MBOAT2, PTCH2, FCGR2A, PXYLP1, YKT6, ABCC5, CDH11, VLDLR, QRFPR, SLC8A3, GABRB2, WFS1, OR2J3, SLC7A8, LRRC4C, PQLC2L, DSE, VIPR2, IL31RA, GLT8D2, PTGIR, UNC5B, NSG1, SMIM10, UNC5C, MCOLN2, MS4A7, KCND1, MRGPRF, MOXD1, NCAM1, GPR55, FBXO18, LSAMP, COL1A1, EMP3, SLITRK5, EMP1, FGFR1, PAM, C3AR1, CAV1, GAL3ST4, HACD1, NKAIN3, EXTL1, B3GNT9, PRUNE2, KCNS2, NDRG4, CRB2, SLC35F1, HEG1, ADAM33, SLC35F4, ENTPD1, CSF1R, HAVCR2, ADAM23, MRC2, ABCB5, SLC4A10, LYVE1, GPR34, FAM198B, DIO3, FREM1, CACNA1G, ADAM22, ANTXR1, CACNA1C, NDST3, NDST4, LRRC8B, LRRC8C, MRVI1, SDC3, TSPAN11, SLC1A5, PCDHGB1, KCNQ4, FRMD5, TMEM173, TSPAN10, RAET1G, TMEM171, EVI2A, GALNTL6, ARHGAP1, ANO4, ATP8B4, NIPAL4, GGTA1P, ADGRE1, PTGER2, PTGER3, ERMN, TMEM255B, ARHGAP28, CHST2, CHST3, LDLRAD3, TMEM244, CHST1, SIGLEC1, TMEM240, CD80, SLC35E4, DPY19L2P1, INPP4B, NRK, JAM2, PRNP, JAM3, C17ORF74, TYRP1, PTCHD1, ADPGK, PPP1R12B, PCDHGC3, GPR1, TMEM159, SCARF2, SNN, MARVELD1, TSPAN32, GPNMB, PLA2R1, MYOF, MS4A6A, SCN5A, HHATL, ZNF804A, MS4A4A, RNF144A, FRZB, TSPAN18, RCAN2, GIMAP1, SLC17A7, TMEM43, SLC6A6, SLC25A53
GO:0005576~extracellular region	*KERA, IL19, SNCA, TGFB3, FGF10, FSTL1, MMP2, CXCL12, IL10, VCL, NOV, APOD, RLN2, COL12A1, BRINP3, CFD, CLCA2, SCUBE3, DRAXIN, OLFML2B, MFGE8, PDGFRL, COL1A2, MFAP2, COL1A1, MFAP4, MFAP5, FGFR1, SCN1B, ELN, PAMR1, CNPY4, TIMP2, IFNA1, HEG1, LAMB1, PLTP, PLAT, ADAM23, PMEL, DEFB136, EFEMP1, EMILIN2, EPHA3, EMILIN1, ELSPBP1, LAMA2, NPS, LAMA4, COL14A1, CXCL14, SFRP1, SFRP2, TLL2, BMP7, BMP5, PLAU, TLL1, FGF9, IGFBP7, RAET1G, RSPO1, XYLT1, LOXL3, SEMA3A, FGF1, LOXL1, IL13RA2, CD200R1, STX1A, ZP1, COL25A1, NLRP3, MMP13, NDNF, SLIT2, MMP11, GRP, SIGLEC1, CTSK, CHRDL1, PLXDC1, VCAN, CHSY1, CNTN4, PLA2G3, ADAM12, LOC389033, CER1, ADAMTS14, ADPGK, TNC, LUM, COL3A1, C1QTNF7, ISLR, CALCB, GLIPR2, COL6A3, COL6A2, COL6A1, PTX3, COL8A1, FIBIN, PLA2R1, COL8A2, THBS2, HAPLN1, COL13A1, ANXA1, NID2, SPARC, FRZB, COL5A2, COL4A6, CLEC11A, FBLN2, IGFBP5*
GO:0005604~basement membrane	*FGF9, TNC, CCDC80, SPARC, NID2, P3H2, LAMA2, LAMA4, FREM1, PTN, LAMB1, LOXL2, THBS2, COL8A2, LOXL1*
GO:0009986~cell surface	*PAM, TMX3, KCNA1, TGFB3, FGF10, L1CAM, LPAR1, TIMP2, SDC3, RGMA, SRPX, HSPA2, ANK3, FAP, TSPAN32, PTN, PLA2R1, CHRNA1, SCN5A, CSF1R, HAVCR2, PLAT, TRPC4, CLMP, SCUBE3, ANXA1, AXL, NLGN2, SPARC, MXRA8, TPBG, SLIT2, ELSPBP1, ADAMTS7, P2RY12, NCAM1, SFRP1, SRPX2, SULF2, CD80, CLIC4, NTRK1, ITGA8, PDGFRB, ANTXR1, PRNP, PLAU, SLC9A1*
GO:0042383~sarcolemma	*SLC8A3, ANXA1, SSPN, LAMA2, ANK1, SGCG, ANK2, ANK3, COL6A3, COL6A2, COL6A1, POPDC2, SCN5A, SGCA*

Table S7. GO Biological processes for *PRRX1* inversely correlated genes

GO Biological processes	Gene Symbols
GO:0055114~oxidation-reduction process	*OXA1L, GMPR2, ALDH1L1, HMGCR, OSGIN1, HIBADH, PECR, AKR7A3, DAO, PCBD2, FAXDC2, CDO1, POR, OGFOD2, SCCPDH, SURF1, HSD17B11, ADHFE1, CYP51A1, FDX1, PAH, FAM213A, GSTK1, MSMO1, PTGR2, CYP2C9, MAOA, MAOB, FOXRED1, PRODH2, DIO1, CYP8B1, DCXR, TM7SF2, ASPDH, CYP2J2, PPOX, CRYL1, AKR1C4, DUS1L, SARDH, COX15, DHCR24, MICAL3, DECR2, CYB5A, GRHPR, C1ORF43, DHRS1, DHRS3, ALDH7A1, DHRS4, CYP27A1, TXNRD2, ALDH9A1, SORD, HSD3B7, DHRS4L2, DHRS4L1, RSAD1, ALDH3A2, PIPOX, FMO4, CYB561D2, FMO5, CBR1, AKR1A1, MMACHC, DHCR7, FMO3, HAAO, HSD17B6, PNPO, DMGDH, CCS, GSTO1, BDH1, HSD17B7, HSD17B8, NSDHL, CHDH, AKR7L, PAOX, DHRS12, DHRS11, CYP4F11, CYP4F12, SOD1, IYD, ADI1, CYP4A11, HSDL2, L2HGDH, SDHC, CYP4F3, AGMO, CYP4F2, CBS, RETSAT*
GO:0006805~xenobiotic metabolicprocess	*ACSM2B, AKR7L, CES2, ACY1, CES1, CYP2J2, CYP2C9, ACY3, EPHX2, RORC, EPHX1, BPHL, POR, CMBL, AADAC, UGT1A6, GLYAT, NR1I2, HNF4A, FMO3, AKR7A3, MGST2*
GO:0006635~fatty acid beta-oxidation	*ECI1, ACOX2, ECI2, ACOX1, ECH1, EHHADH, ECHS1, PEX5, ECHDC2, ACAT2, PEX7, ABCD3, HIBCH, HSD17B4, ACAD11, ACAA1*
GO:0008152~metabolic process	*ARSE, ECH1, AQP9, EHHADH, ECHDC2, ECHDC3, AFMID, UGT1A6, GSTO1, AGPAT3, ENOSF1, ECI1, ACSM2B, GSTA1, ECI2, CES1, ACY3, ACSM2A, EPHX2, ISOC1, GRHPR, OXSM, AADAC, CENPV, ARSA, HDHD3, FLAD1, SCP2, ACSM5, ACAA1*
GO:0042632~cholesterol homeostasis	*TMEM97, SLC37A4, EPHX2, MTTP, ABCG8, APOB, APOA2, APOA1, ABCG5, DGAT2, XBP1, APOC3, PCSK9, SCARB1, ANGPTL3, LIPC, APOM, NR1H3*
GO:0007031~peroxisome organization	*PEX11A, SEC16B, TMEM135, PEX1, PEX19, PEX16, PEX6, ABCD3, SCP2, PEX7*
GO:0006633~fatty acid biosynthetic process	*ACSM2B, MSMO1, HNF1A, FAXDC2, ACSM2A, PRKAB2, OXSM, PECR, XBP1, ABCD3, AGMO, LIPC, PCCB, ACSM5, HSD17B8*
GO:0006695~cholesterol biosyntheticprocess	*TM7SF2, EBP, MSMO1, APOA1, CES1, HMGCR, CYP51A1, DHCR7, FDPS, PMVK, HSD17B7, NSDHL, DHCR24*
GO:0006699~bile acid biosynthetic process	*ACOX2, HNF1A, AKR1C4, CYP27A1, ABCB11, HSD3B7, HSD17B4, CYP8B1, SCP2, SLC27A5*
GO:0006631~fatty acid metabolic process	*ACSM2B, PPARA, CYP4A11, CRYL1, ACADSB, MSMO1, C3, EHHADH, ACSM2A, LYPLA2, ANGPTL3, GPAM, ACSM5, RPP14*

Table S8. GO Cellular components for *PRRX1* inversely correlated genes

GO Cellular components	Gene Symbols
GO:0005739~mitochondrion	*MRPL40, TUSC2, OXA1L, MRPS33, ALDH1L1, TARS2, PSTK, NIT1, NIT2, EHHADH, MALSU1, BPHL, IARS2, GLYATL1, HIBADH, PECR, SLC25A20, CRY2, CISD3, TCAIM, TFB2M, DDAH1, HMGCL, ACSM2B, CRLS1, GATM, RPUSD2, PCBD2, ACSM2A, SECISBP2, KIAA0141, TMEM126A, CECR5, POR, SCCPDH, MRPS18B, NBR1, MRPL49, ABAT, ATPIF1, AKAP1, PCCB, MRPL46, MPST, ACADSB, ADHFE1, HACD3, HAX1, FDX1, ALDOC, ACP6, SFXN1, AGMAT, MTIF2, ACAT2, GFM2, GCKR, TOMM6, MTCH2, FAM213A, IVD, GSTK1, IDH1, LINC00493, SUGCT, FH, MRPS27, NADK2, MRPS22, TP53BP2, MAOA, MAOB, FDPS, C21ORF33, JTB, MPC2, CRAT, SPRYD4, TST, GLYCTK, NMT1, POLRMT, SLC25A13, PTCD1, NIPSNAP1 HDHD3, PRODH2, FOXRED1, COQ10A, THNSL1, ATP7B, DAP3 ACOX2, ACOX1, BCKDK, COX11, COA3, AMT, HINT2, GTPBP10, BNIP3, PEX5, ECHDC2, ECHDC3, PRDX3, TRIAP1, ALAS1, VWA8, NARS2, MPV17L, GNPAT, ACOT13, CAT, DHTKD1, ACAD9, CDK5RAP1, SARDH, HEMK1, COX15, SUOX, ALDH5A1, OTC, COQ5, ALDH7A1, DHRS4, DGAT2, TSTD1, TOMM20, TXNRD2, AARS2, USP30, ECH1, ABHD6, ADH5, NFS1, ECHS1, RSAD1, TACO1, RPP14, PLIN5, MMACHC, HSD17B4, SLC25A42, BDH1, ACSL5, ENOSF1, ECI1, SHMT1, ECI2, RMDN1, SIRT4, SIRT5, PCK2, SOD1, PHYH, OXSM, SIRT3, MRPL24, GLYAT, SMDT1, L2HGDH, HSDL2, SDHC, POLDIP2, BOLA1, HIBCH, SCP2*
GO:0005777~peroxisome	*ACOX2, ACOX1, HACL1, ECH1, EHHADH, PEX6, PEX5, PMVK, AGXT, PEX11G, ALDH3A2, ACOT4, PIPOX, PEX7, PECR, PEX19, PEX1, MPV17L, GSTK1, PEX16, PXMP4, GNPAT, ABCD3, IDH1, DAO, CAT, HSD17B4, HMGCL, NUDT12, EPHX2, CRAT, ISOC1, SOD1, PHYH, HAO1, PEX11A, TMEM135, DHRS4, HSDL2, ACAD11, SCP2, ACAA1*
GO:0005789~endoplasmic reticulummembrane	*RFT1, HMGCR, C14ORF1, APOB, PGRMC1, ELOVL2, CREB3L4, SLC51A, SAR1B, TMEM203, DDRGK1, FAXDC2, SMIM14, MOGS, YIF1A, PNPLA3, POR, ERGIC3, AADAC, EBPL, GGCX, SYVN1, CYP51A1, CERS4, ANKS4B, LMAN2, MIA3, CERS2, ZDHHC9, PEMT, REEP6, MSMO1, SEC11C, CYP2C9, EPHX1, TMEM110, EI24, TXNDC11, RNF5, FAAH, SPCS1, DIO1, CYP8B1, ALG14, TM7SF2, SEC24A, CYP2J2, ATL2, ALG3, ALG5, ALG6, ALG8, ALG9, PIGM, RNF103, SLC35D1, POMT1, PCYT2, AGPAT3, DHCR24, PGAP2, STX5, EVA1A, PIGV, CYB5A, SIGMAR1, PIGP, SCAP, DHRS3, DHRS4, ZDHHC16, DGAT2, ORMDL2, UGT2B10, ORMDL3, STBD1, DOLPP1, SLC27A5, SERP1, APH1A, HSD3B7, SLC37A4, TMEM259, ALDH3A2, SEC16B, FMO4, UGT1A6, FMO5, TMED5, TMED2, XBP1, DHCR7, FMO3, PEX16, HSD17B7, ACSL5, NSDHL, MOGAT3, EBP, NAT8, HPN, TMBIM6, CYP4F11, CYP4F12,*

	GJB1, ITPR2, CYP4A11, RNF43, SDF2L1, CYP4F3, SVIP, AGMO, CYP4F2, ABCC6, MGST2, RETSAT
GO:0005782~peroxisomal matrix	*ACOX2, ACOX1, ECI2, HACL1, PAOX, NUDT12, EHHADH, PEX5, CRAT, GRHPR, ACOT6, AGXT, PHYH, ACOT4, PIPOX, PEX7, HAO1, GNPAT, ABCD3, IDH1, DAO, CAT, HSD17B4, SCP2, ACAA1*
GO:0005759~mitochondrial matrix	*BCKDK, IBA57, TARS2, BTD, MALSU1, AMT, BPHL, IARS2, PRDX3, AGXT, HIBADH, ALAS1, MCEE, NARS2, TFB2M, DHTKD1, SARDH, HMGCL, ACSM2B, SUOX, ALDH5A1, OTC, ACSM2A, HAGH, ALDH7A1, HYKK, CYP27A1, TXNRD2, ABAT, FLAD1, PCCB, PMPCB, ACADSB, ADHFE1, NAGS, FDX1, NFS1, ECHS1, MTHFS, GFM2, IVD, GSTK1, DMGDH, BDH1, HSD17B8, FH, ECI1, SIRT4, SIRT5, PCK2, SOD1, MMAB, SIRT3, TST, POLRMT, GLYAT, PTCD1, ALDH2, HIBCH, ACSM5*
GO:0005778~peroxisomal membrane	*ACOX1, HMGCR, DECR2, PEX6, PEX5, ALDH3A2, PEX11A, PECR, DHRS4, PEX19, PEX1, MPV17L, PXMP4, PEX16, GNPAT, ABCD3, PXMP2, DAO, CAT, HSD17B4*
GO:0070062~extracellular exosome	*RARRES2, ALAD, CADM1, BTD, MASP2, PPCS, SELENBP1, FAH, ATP2B2, APOB, WWP1, APOH, APOM, DDAH1, F12, GATM, ABCB11, CDHR5, TAF6L, SERPINF2, F2, PGM1, RAB17, ABAT, EPS8L2, MPST, ACADSB, AHCY, SERPINA10, ALDOC, MST1, ACAT2, AHSG, HNMT, RAC3, FAM213A, KLKB1, IDH1, FH, COL18A1, C11ORF52, BHMT2, ST6GAL1, C11ORF54, PTGR2, CFB, GUSB, UPB1, MAOB, CBLC, PON1, TPRG1L, QPRT, PDZK1, PON3, CYP2J2, ARSE, TOLLIP, PMVK,SLC23A1, SERPINA6, SERPINA5, SERPINA4, SLC25A1, CEACAM1, DPP4, HYAL1, RAB4A, TTC38, CYB5A, GRHPR, NAPRT, AMBP, DNPH1, TF, SORD, ECH1, C3, ABHD6, DPYS, SEC14L2, CPN2, STAU1, GALK1, CBR1, AGT, SERPINC1, HAAO, C2, MYO5B, ECI1, AKR7L, CES3, SHMT1, NAT8, CES2, FTCD, SOD1, PCK2, PLG, KHK, TOM1L1, ARSA, SVIP, GAMT, SERPIND1, EIF6, ALDH1L1, NIT1, THRB, NIT2, IQGAP2, BPHL, VTN, CMBL, AZGP1, TTR, APOA2, NUDCD2, APOA1, PGRMC1, ST3GAL6, CTDSP1, DERA, AKR7A3, CUTA, TMEM205, NUDT5, PDXP, LYPLA2, RBKS, MOGS, METTL7A, IGSF8, NBR1, ACTR3B, PAH, LMAN2, GIPC2, AGMAT, GALM, MTCH2, ALB, GSTK1, GMPPA, APOC3, ENTPD5, GALE, SCARB1, P4HB, ACY1, B4GAT1, ACY3, MYO1B, EPHX2, SPPL2A, CRB3, ABHD14B, RFNG, ABCB4, TST, APEH, ALDH2, MYH14, CPB2, DCXR, HINT1, PRDX3, SDC4, LSR, CRYL1, AKR1C4, PEX1, ITIH1, ACOT13, ITIH2, ITIH3, CAT, KNG1, DDC, HAGH, ALDH7A1, DHRS4, PKLR, DDT, PEBP1, TOM1, CUX2, ALDH9A1, XYLB, PRKCZ, GPRC5C, ADH5, ADH6, ECHS1, OAF, ALDH3A2, DDTL, UGT1A6, TEX264, AKR1A1, PNPO, GSTO1, SLC39A5, GBA, GSTA1, COBLL1, HPN, CPPED1, FETUB, ISOC1, SCRN2, A1BG, CYP4A11, GLYAT, MPI, HIBCH, SCP2, VPS25*
GO:0005743~mitochondrial innermembrane	*MRPL40, COX11, OXA1L, MRPS33, SLC25A20, ATP5S, ABCB10, SLC25A1, ACAD9, HMGCL, OMA1, COX15, MRPL2, CRLS1, GATM, OTC, NDUFC2, TMEM126A, DHRS1, CYP27A1,*

	MRPS18B, MRPL49, GPAM, SURF1, MRPL46, SFXN4, SFXN1, SFXN2, ALDH3A2, MRPL11, MTCH2, GSTK1, MRPL16, ABCD3, SLC25A44, SLC25A42, ACSL5, ECI1, MRPS27, CHDH, MRPS22, MAOB, SIRT4, SIRT5, MPC2, CRAT, SIRT3, MRPL24, TST, SLC25A13, L2HGDH, SDHC, SLC25A10, NIPSNAP1, PRODH2, FOXRED1, COQ10A, ACAD11, DAP3
GO:0031090~organelle membrane	*TM7SF2, CYP2J2, SEC11C, CYP2C9, CYP51A1, EPHX1, CYP4F11, CYP4F12, FMO4, AADAC, FMO5, CYP4A11, PGRMC1, FAAH, FMO3, SPCS1, CYP4F3, CYP4F2, UGT2B10, CYP8B1, MGST2*
GO:0005783~endoplasmic reticulum	*TM7SF2, ATL2, HMGCR, ALG3, BNIP3, ALG9, ATP2B2, RNF103, AQP11, POMT1, PGRMC1, ELOVL2, CREB3L4, CAT, SAR1B, AGPAT3, LINC01547, DHCR24, TMEM203, DDRGK1, SMIM14, CCDC47, CNPY2, MOGS, SIGMAR1, METTL7A, PROC, SCAP, DHRS1, EBPL, DGAT2, ORMDL2, ORMDL3, TMCO1, SLC27A5, SERP1, FGFR4, SYVN1, APH1A, HAX1, HACD3, SLC38A9, CYP51A1, SLC37A4, CERS4, NHLRC1, CLDN14, STAU1, FMO5, UGT1A6, A1CF, TMED2, ALB, XBP1, CERS2, DHCR7, RNF128, ZDHHC9, PEX16, ENTPD5, HSD17B6, PCSK9, PCSK6, HSD17B7, ACSL5, NSDHL, EBP, P4HB, CES2, MSMO1, TMBIM6, FTCD, CRAT, MTTP, EI24, TXNDC11, AGMO, POFUT1, MGST2*

Table S9. Metabolic targets in the *PRRX1* co-expressed gene list

In HCC	Gene Symbol
Up (n=11)	*ALG3, FLAD1, COX11, FDPS, NAT9, ALG6, PPOX, GNPAT, SLC35B1, ALG8, MPC2*
Down (n=124)	*A1CF, ABAT, ABCA6, ABCC6, ABCD3, ABHD6, ACAA1, ACADSB, ACBD4, ACOX1, ACOX2, ACSL5, ACSM5, ADH6, ADI1, AGXT, AKR7A3, ALAS1, ALDH1L1, ALDH2, ALDH7A1, ALDH9A1, AQP9, BDH1, BHMT2, CAT, CBS, CDO1, CES1, CES3, CRAT, CRYL1, CYB5A, CYP2C9, CYP2J2, CYP4A11, CYP4F12, CYP4F2, CYP4F3, DAO, DCXR, DDAH1, DDT, DERA, DHRS1, DHRS12, DHTKD1, DPYS, ECHDC2, ECHDC3, ECHS1, EHHADH, EPHX2, FAAH, FAH, FDX1, FMO3, FMO4, FTCD, GAMT, GATM, GLYAT, GNE, GPHN, GRHPR, GSTA1, HAAO, HAGH, HAO1, HIBCH, HMGCL, HNMT, HSD17B6, HYAL1, IVD, KHK, LIPC, MAOB, MAT1A, MGST2, MMACHC, MTHFS, MTTP, NIT2, OTC, PAH, PAOX, PCCB, PCK2, PECR, PEMT, PFKFB1, PGM1, PHYH, PIGV, PIPOX, PON1, PON3, PRODH2, QPRT, RBKS, RETSAT, SARDH, SCP2, SFXN1, SHMT1, SLC10A1, SLC17A2, SLC25A20, SLC27A5, SLC2A2, SLC37A4, SLC47A1, SLC6A12, SOD1, SORD, ST3GAL6, ST6GAL1, SULT2A1, SUOX, TF, THNSL1, UAP1, UPB1*

Table S10. Genes differentially expressed in *PRRX1*-high tumours relative to *PRRX1*-low tumours (attached as a separate Excel file)

Table S11.148 genes as potential candidates that likely cooperate or are coregulated with *PRRX1*

Genes involved in	Gene symbol
EMT	*CDH1, MMP2, MMP9, SNAIL, TWIST, VIM, ZEB1, ZEB2*
Recurrence of HCC	*ATP7B, FAH, HNF1A, IL10, PNPLA3*
Metabolism	*A1CF, ABAT, ABCA6, ABCC6, ABCD3, ABHD6, ACAA1, ACADSB, ACBD4, ACOX1, ACOX2, ACSL5, ACSM5, ADH6, ADI1, AGXT, AKR7A3, ALAS1, ALDH1L1, ALDH2, ALDH7A1, ALDH9A1, ALG3, ALG6, ALG8, AQP9, BDH1, BHMT2, CAT, CBS, CDO1, CES1, CES3, COX11, CRAT, CRYL1, CYB5A, CYP2C9, CYP2J2, CYP4A11, CYP4F12, CYP4F2, CYP4F3, DAO, DCXR, DDAH1, DDT, DERA, DHRS1, DHRS12, DHTKD1, DPYS, ECHDC2, ECHDC3, ECHS1, EHHADH, EPHX2, FAAH, FAH, FDPS, FDX1,FLAD1, FMO3, FMO4, FTCD, GAMT, GATM, GLYAT, GNE, GNPAT, GPHN, GRHPR, GSTA1, HAAO, HAGH, HAO1, HIBCH, HMGCL, HNMT, HSD17B6, HYAL1, IVD, KHK, LIPC, MAOB, MAT1A, MGST2, MMACHC, MPC2, MTHFS, MTTP, NAT9, NIT2, OTC, PAH, PAOX, PCCB, PCK2, PECR, PEMT, PFKFB1, PGM1, PHYH, PIGV, PIPOX, PON1, PON3, PPOX, PRODH2, QPRT, RBKS, RETSAT, SARDH, SCP2, SFXN1, SHMT1, SLC10A1, SLC17A2, SLC25A20, SLC27A5, SLC2A2, SLC35B1, SLC37A4, SLC47A1, SLC6A12, SOD1, SORD, ST3GAL6, ST6GAL1, SULT2A1, SUOX, TF, THNSL1, UAP1, UPB1*

Table S12. *ZEB1* expression in combination with *PRRX1* with respect to the clinicopathological variables (n= number of patients)

**Characteristics**	PRRX1 ↑ ZEB1↓ n	PRRX1 ↓ ZEB1↑ n	P-value
**ALT**			
Low	36	36	ns
High	21	21	
**Tumor size**			
Small	35	35	ns
Large	22	22	
**Cirrhosis**			
Yes	52	55	ns
No	5	2	
**TNM staging**			
I	27	23	ns
II	18	20	
III	11	14	
**BCLC**			
0	9	6	ns
A	40	35	
B	4	8	
C	3	8	
**AFP**			
Low	29	27	ns
High	26	30	

Table S13. *ZEB2* expression in combination with *PRRX1* with respect to the clinicopathological variables (n= number of patients)

**Characteristics**	PRRX1 ↑ ZEB2↓ n	PRRX1 ↓ ZEB2↓ n	*P*-value
**ALT**			
Low	32	28	ns
High	24	27	
**Tumor size**			
Small	37	36	ns
Large	19	19	
**Cirrhosis**			
Yes	52	53	ns
No	4	2	
**TNM staging**			
I	23	23	ns
II	18	20	
III	14	12	
**BCLC**			
0	6	3	ns
A	37	37	
B	2	8	
C	10	7	
**AFP**			
Low	31	30	ns
High	25	25	

Supplementary References

1. Nwosu ZC, Megger DA, Hammad S, Sitek B, Roessler S, Ebert MP et al., Identification of the Consistently Altered Metabolic Targets in Human Hepatocellular Carcinoma. Cellular and Molecular Gastroenterology and Hepatology. **2017**;4(2):303-23. doi: 10.1016/j.jcmgh.2017.05.004.
2. Ocana OH, Corcoles R, Fabra A, Moreno-Bueno G, Acloque H, Vega S, et al. Metastatic colonization requires the repression of the epithelial-mesenchymal transition inducer Prrx1. Cancer cell. **2012**;22(6):709-24. doi: 10.1016/j.ccr.2012.10.012.
